# Position-specific intron retention is mediated by the histone methyltransferase SDG725

**DOI:** 10.1186/s12915-018-0513-8

**Published:** 2018-04-30

**Authors:** Gang Wei, Kunpeng Liu, Ting Shen, Jinlei Shi, Bing Liu, Miao Han, Maolin Peng, Haihui Fu, Yifan Song, Jun Zhu, Aiwu Dong, Ting Ni

**Affiliations:** 1 State Key Laboratory of Genetic Engineering and MOE Key Laboratory of Contemporary Anthropology, Collaborative Innovation Center of Genetics and Development, Human Phenome Institute, School of Life Sciences and Huashan Hospital, Fudan University, Shanghai, 200438 People’s Republic of China; 2 State Key Laboratory of Genetic Engineering, Collaborative Innovation Center of Genetics and Development, International Associated Laboratory of CNRS-Fudan-HUNAU on Plant Epigenome Research, Department of Biochemistry, Institute of Plant Biology, School of Life Sciences, Fudan University, Shanghai, 200438 People’s Republic of China; 3 Systems Biology Center, National Heart Lung and Blood Institute, National Institutes of Health, Bethesda, MD 20892 USA

**Keywords:** Intron retention, Histone H3K36 methylation, Shift, SDG725, Rice

## Abstract

**Background:**

Intron retention (IR), the most prevalent alternative splicing form in plants, plays a critical role in gene expression during plant development and stress response. However, the molecular mechanisms underlying IR regulation remain largely unknown.

**Results:**

Knockdown of SDG725, a histone H3 lysine 36 (H3K36)-specific methyltransferase in rice, leads to alterations of IR in more than 4700 genes. Surprisingly, IR events are globally increased at the 5′ region but decreased at the 3′ region of the gene body in the *SDG725*-knockdown mutant. Chromatin immunoprecipitation sequencing analyses reveal that SDG725 depletion results in a genome-wide increase of the H3K36 mono-methylation (H3K36me1) but, unexpectedly, promoter-proximal shifts of H3K36 di- and tri-methylation (H3K36me2 and H3K36me3). Consistent with the results in animals, the levels of H3K36me1/me2/me3 in rice positively correlate with gene expression levels, whereas shifts of H3K36me2/me3 coincide with position-specific alterations of IR. We find that either H3K36me2 or H3K36me3 alone contributes to the positional change of IR caused by *SDG725* knockdown, although IR shift is more significant when both H3K36me2 and H3K36me3 modifications are simultaneously shifted.

**Conclusions:**

Our results revealed that SDG725 modulates IR in a position-specific manner, indicating that H3K36 methylation plays a role in RNA splicing, probably by marking the retained introns in plants.

**Electronic supplementary material:**

The online version of this article (10.1186/s12915-018-0513-8) contains supplementary material, which is available to authorized users.

## Background

Intron retention (IR), a specific form of pre-messenger RNA (pre-mRNA) alternative splicing (AS), has attracted increasing attention given its role in global gene expression regulation in both animals and plants [1–6]. Notably, IR is the most prevalent form of AS in higher plants, possibly due to the shorter intron length in plants than in animals [7, 8]. Genome-wide analyses revealed that greater than half of the AS events in rice belong to IR [9, 10].

Several studies have highlighted the functional importance of IR in plants. For example, IR has been shown to associate with abiotic stress response in barley [5, 11]. In strawberry, the level of IR is significantly reduced in post-fertilization compared to pre-fertilization, suggesting the involvement of IR in fruit maturation [12]. Retention of an intron in the 5′ UTR of the Zinc-Induced Facilitator 2 gene (*ZIF2*) enhances zinc tolerance in Arabidopsis [6]. Two intron-retained transcripts in Arabidopsis have been shown to remain in the nucleus to avoid nonsense-mediated degradation (NMD) [13]. During Arabidopsis gametophyte development, IR regulates translation in a transcription-independent and spliceosome-dependent manner [14]. Taken together, all these findings underscore the importance of IR in plant growth and development.

Epigenetic regulators are known to be involved in AS regulation [4, 15, 16]. Histone modifications are particularly interesting because of their potential links between chromatin structure and co-transcriptional pre-mRNA splicing. Chromatin immunoprecipitation sequencing (ChIP-seq) analyses in human and mouse cells showed that H3K36me3 (tri-methylation of histone H3 at lysine 36) signals in introns and alternatively spliced exons are considerably lower than those in constitutive exons, suggesting that H3K36me3 modification likely acts as a mark for exons in animal cells [17]. Furthermore, H3K36me3 participates in AS by recruiting the splicing factor polypyrimidine tract-binding protein (PTB) via MRG15, an H3K36me3 reader protein in human [16]. BS69, a specific reader protein for H3.3K36me3, is involved in pre-mRNA splicing, especially IR, by interaction with the U5 small cytoplasmic fractionation extraction ribonucleoprotein (snRNP) in human cells [4]. Pajoro et al. discovered that H3K36me3 played a role in AS and flowering control in Arabidopsis*,* wherein mutants of SDG8 and SGD26, two methyltransferases of H3K36, affect temperature-dependent flowering [18]. All these findings support the notion that H3K36me3 plays a direct role in regulating AS.

However, it remains unclear whether all three forms of H3K36 methylation (mono-, di-, and tri-) are involved in AS regulation, especially IR, in plants. We previously reported that two H3K36-specific methyltransferases, SDG725 and SDG708, modulate gene transcription and affect rice growth and development [19–21]. In this study, we investigated splicing alterations in the *SDG725*- and *SDG708-*knockdown rice mutants by RNA sequencing (RNA-seq). We found that knockdown of *SDG725* led to altered IR in thousands of genes. In addition, IR events tend to increase at the 5′ portion but decrease at the 3′ part of the gene body when comparing the *SDG725*-knockdown mutant to the wild-type (WT) plants. The results coincided with a higher H3K36me2 occupancy at the 5′ part but a lower one at the 3′ part of the gene body. In contrast, *SDG708* knockdown did not cause either these promoter-proximal shifts of IR or histone modification. Our work discovered a previously unknown shift of IR and its possible epigenetic regulator in rice.

## Results

### SDG725 regulates a global shift of intron retention in rice

Since H3K36 methylation has been proposed for splicing regulation in animals, we sought to investigate whether a similar mechanism is also employed in plants. We took advantage of two transgenic rice lines previously generated, in which *SDG725* or *SDG708* was efficiently knocked down by RNA interference [19, 21]. Quantitative mass spectrometry showed that knockdown of *SDG725* led to an increased level of H3K36me1 modification, but decreased levels of both H3K36me2 and H3K36me3 (Additional file 1: Figure S1) [12]. Two biological replicates of RNA-seq libraries were constructed, sequenced, and analyzed for *725Ri-1* (a stable RNAi line of *SDG725*)*, 708Ri-1* (a stable RNAi line of *SDG708*), and WT rice plants (Additional file 2: Table S1) [19, 21]. As the result of *SDG725* knockdown, RNA-seq analyses revealed that 462 and 496 genes were up- and down-regulated, respectively (Additional file 1: Figure S2a). Gene ontology analysis showed that the differentially expressed genes (DEGs) in *725Ri-1* are enriched in metabolic and biosynthetic processes (Additional file 1: Figure S2b). The DEGs in *708Ri-1* (245 up- and 222 down-regulated) also are enriched in metabolic processes, but only a small fraction of them overlapped with those found in *725Ri-1* mutant plants [21], indicating the distinct biological roles of these two H3K36-specific methyltransferases in rice.

**Fig. 1:**
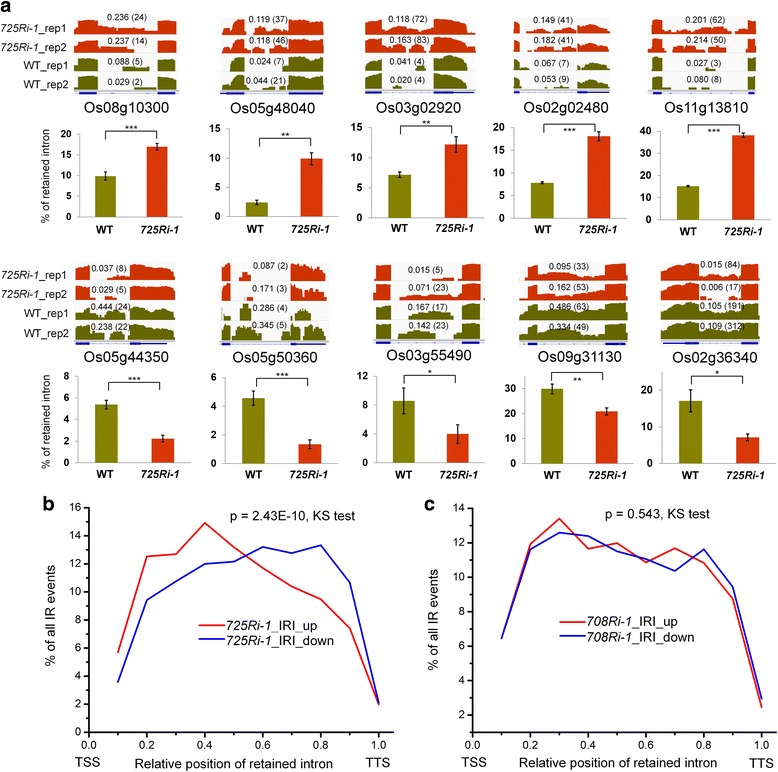
SDG725, but not SGD708, affects global intron retention shift. **a** RNA-seq tracks (log2 transformed) and qRT-PCR validation for 10 differentially retained introns between *725Ri-1* and WT plants. Two biological replicates (*rep1* and *rep2*) of RNA-seq data of *725Ri-1* and WT are shown. IRI and read numbers (*in parentheses*) are shown in the corresponding introns. Rice genes are shown below RNA-seq tracks. *, **, and *** indicate *p* values < 0.05, < 0.01, and < 0.001, respectively (*t* test). **b**, **c** Distribution of up-regulated (*red*) and down-regulated (*blue*) intron retention (IR) events between *725Ri-1* and wild-type (*WT*) rice (**b**), and between *708Ri-1* and WT (**c**). A twofold change in IRI was applied as a cutoff to define differential IR events. The retained introns also must be supported by at least 3 reads, length coverage ≥80%, and host gene expression ≥1 FPKM. Average IRI value of two biological replicates of both WT and mutant was used for location distribution Kolmogorov-Smirnov (*KS*) test. *TSS* transcription start site, *TTS* transcription termination site

The splicing changes in rice were further analyzed by the SplAdder approach [22]. Consistent with previous findings [23], IR is the predominant form of AS events identified by comparing to rice genome annotation (Additional file 2: Table S2). To perform a quantitative investigation on IR, we used the intron retention index (IRI) to estimate the extent of retention for each annotated intron [3]. We found that 4714 genes contain one or more altered IR, including 2089 IRI-up introns (≥ twofold increase in IRI) and 4214 IRI-down introns (≥ twofold decrease in IRI) by comparing the *725Ri-1* plants to the WT rice plants. Ten IRI-altered introns were selected for validation by reverse transcription followed by quantitative polymerase chain reaction (qRT-PCR). Primer pairs were designed to detect either spliced or intron-retained transcripts for each IR event (Additional file 2: Table S3). For all 10 cases, qRT-PCR results confirmed the RNA-seq data (Fig. 1a). To our surprise, we found that the IRI-up introns were favored at the 5′ portion of the gene body while the IRI-down introns were preferred at the 3′ portion of the gene body when comparing *725Ri-1* to WT rice plants using stringent coverage requirements (Fig. 1b**,** Additional file 1: Figure S3). The conclusion remained the same when the requirements of intron length coverage were relaxed (Additional file 1: Figure S4). In contrast, no location bias was observed between the 3326 IRI-up and 2497 IRI-down introns in *708Ri-1* (Fig. 1c**,** Additional file 2: Table S4). For exon skipping, no obvious position-specific splicing alteration was found by comparing *725Ri-1* with WT rice. Our results showed that knockdown of SDG725 but not SDG708 alters IR in a position-specific manner.

### Genes with increased IR show reduced expression levels

**Fig. 2:**
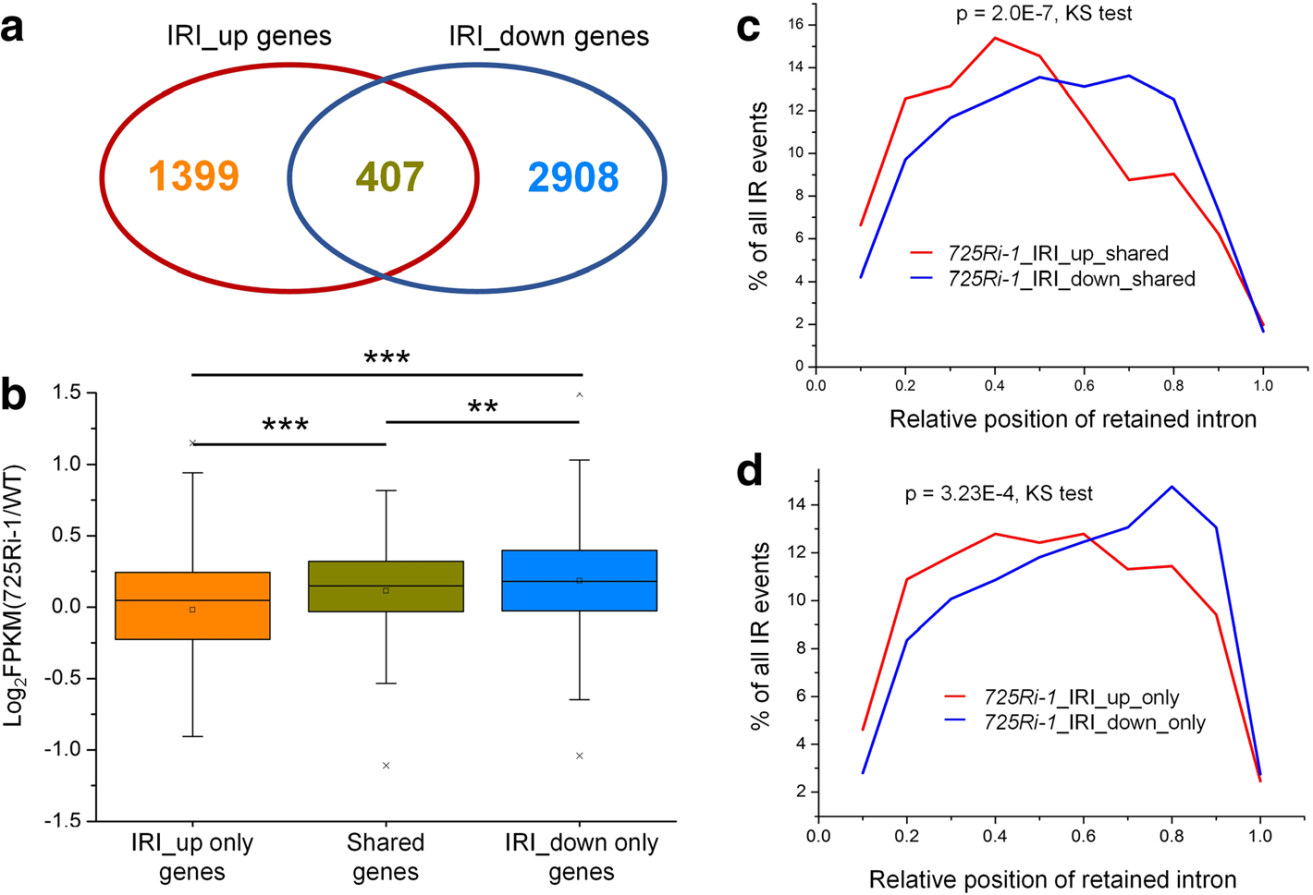
Characterization of genes with changed IR events between *725Ri-1* and WT rice. **a** The Venn diagram denotes the relationship between genes with up- and down-regulated IR events. **b** Box plot of gene expression differences between *725Ri-1* and wild-type (WT) rice for the three types of genes. ** and *** indicate *p* values < 0.01 and < 0.001, respectively (*t* test). **c**, **d** Distribution of up- (*red*) and down-regulated (*blue*) IR events in the 407 genes with both intron retention index (IRI)-up and IRI-down introns (**c**) or in genes with only IRI-up or IRI-down introns (**d**). A twofold change in IRI was applied as a cutoff to define up- or down-regulation

Among all the genes with one or more retained introns, 1399 genes only contain IRI-up introns, 2908 genes only contain IRI-down introns, and 407 genes contain both IRI-up and IRI-down introns (Fig. 2a). IR may regulate gene expression through several different mechanisms, including but not limited to the following: (1) intron-retained transcripts are unstable, being degraded by nuclear RNA surveillance machinery or cytoplasmic NMD [3, 13, 24–26]; (2) intron-retained transcripts are stable and can be translated into new protein variants, where those introns are known as “exitrons” [27–29]; (3) a retained intron in a 5′ UTR regulates translation initiation [30] while IR in a 3′ UTR can either repress mRNA stability and translation by introducing more regulatory *cis* elements [31] or stabilize mRNA by avoiding NMD [32]. Therefore, global IR coupled with RNA stability could serve as a novel mechanism to fine-tune gene expression in rice. RNA-seq data were then used to determine the effect of altered IR on gene expression. We found that the expression level of the genes with only IRI-down introns tends to be up-regulated compared to those with only IRI-up introns, and the expression changes of the 407 genes with both IRI-up and IRI-down introns fall in between (Fig. 2b). These observations agreed with the notion that IR may serve as a post-transcriptional mechanism to reduce gene expression [1–4]. Consistent with Fig. 1b, in the 407 genes, IRI-up introns show a similar preference at the 5′ part of the gene body and IRI-down introns are enriched at the 3′ end of the gene body (Fig. 2c). The same occurs with the genes with IRI-up only and IRI-down only introns (Fig. 2d).

### Intron retention shift correlates with distribution shifts of H3K36 methylations

**Fig. 3:**
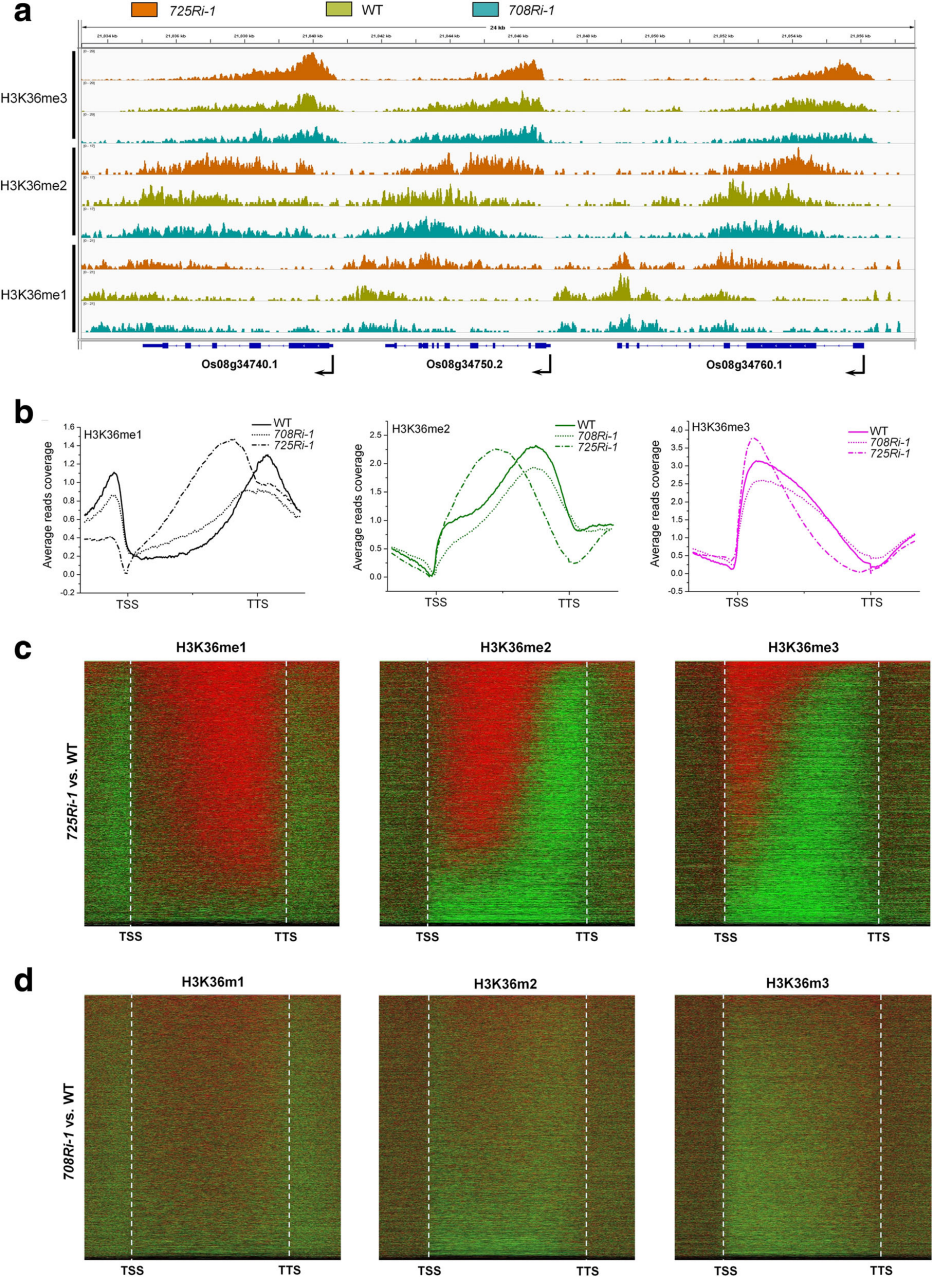
Knockdown of *SDG725*, not *SDG708*, causes pattern shifts of H3K36 methylations in rice. **a** An Integrative Genomics Viewer display of H3K36me1, H3K36me2, and H3K36me3 profiles for a gene-rich region in *725Ri-1, 708Ri-1*, and WT rice plants. **b** The distribution patterns of H3K36me1 (*left*), H3K36me2 (*middle*), and H3K36me3 (*right*) in *725Ri-1*, *708Ri-1*, and WT rice plants, respectively. **c**, **d** Differential H3K36 methylation analysis between *725Ri-1* and WT plants (**c**) or between *708Ri-1* and WT rice plants (**d**). Each line in a heatmap represents a gene. The increased and decreased ChIP-seq signals (mutant vs. WT) are shown in *red* and *green*, respectively. Genes are sorted in descending order based on the number of red bins in the gene body (see details in Methods)

We next asked why IR is shifted as the result of *SDG725* knockdown. Considering the role of H3K36 methylation in IR in animals, we acquired H3K36 mono-, di-, and tri-methylation (H3K36me1/me2/me3) profiles of the *725Ri-1*, *708Ri-1*, and WT rice plants using ChIP-seq (Additional file 2: Table S5). By looking at gene-dense regions we found that H3K36me2 and H3K36me3 profiles exhibit apparent promoter-proximal shifts in *725Ri-1* but not in *708Ri-1* compared to those in WT rice plants (Fig. 3a). These observations were further confirmed by genome-wide analyses (Fig. 3b–d). It is worth noting that H3K36me1 levels are increased in nearly the entire gene body (Fig. 3b**,** left panel) upon *SDG725* knockdown. In contrast, *SDG708* knockdown led to a global decrease of all three H3K36 methylation marks (Fig. 3a, b).

To obtain a detailed view of H3K36 methylation changes at the individual gene level, we computed the difference of ChIP-seq tag density gene by gene between knockdown and WT plants (see details in Methods, Additional file 1: Figure S5). For almost all transcribed loci in the *725Ri-1* plants, H3K36me1 levels are increased across the gene body (Fig. 3c, left panel), while H3K36me2 (Fig. 3c, middle panel) and H3K36me3 (Fig. 3c, right panel) levels are increased at the 5′ end but decreased at the 3′ end of the gene body. However, this histone methylation positional bias was not detected in the *708Ri-1* plants (Fig. 3d), suggesting that the promoter-proximal shifts of H3K36me2/me3 are specific for the *725Ri-1* plants.

### H3K36me2/me3 shifts positively correlate with IR shifts caused by *SDG725* knockdown

**Fig. 4:**
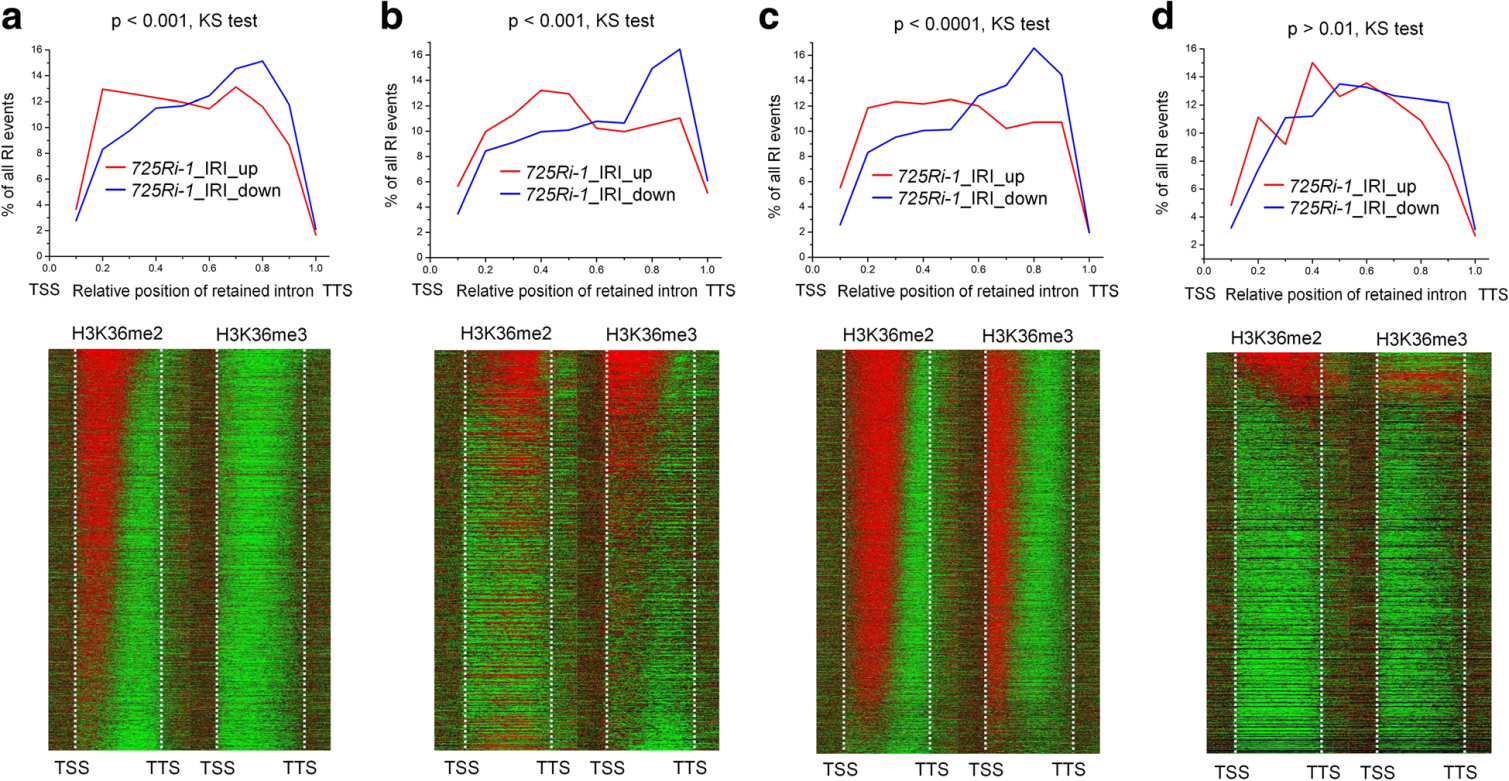
H3K36me2 and H3K36me3 contribute to the global shift of intron retention. Distribution of up- (*red*) and down-regulated (*blue*) retained intron (*RI*, twofold) in genes with a shifted H3K36me2 profile (**a**), a shifted H3K36me3 profile (**b**), both (**c**), or neither (**d**). IR distributions and heatmaps of H3K36 methylations are shown in the *top* and *bottom panels*, respectively. Each line of a heatmap represents a gene, and the tag counts in the gene body were divided into 300 bins. Increased and decreased ChIP-seq signals (*725Ri-1* vs. WT) are shown in *red* and *green*, respectively. Genes are sorted in descending order based on the number of red bins in the gene body. See Methods for details of how to define H3K36me2/me3 shifted genes

*SDG725* knockdown caused global shifts of H3K36me2 and H3K36me3, providing us with a unique opportunity to examine the potential contributor for IR shift. We therefore divided the transcribed genes into four groups. For type I genes, only H3K36me2 show a promoter-proximal shift. Type II genes are designated for H3K36me3 shift towards the 5′ end, while type III genes exhibit pattern shifts for both H3K36 methyl marks. Type IV genes show no shift for either H3K36me2 or H3K36me3. We noticed that H3K36me2 shift alone led to a considerable effect on the location bias of IR events (Fig. 4a). Similarly, H3K36me3 shift alone has an impact on IR shift (Fig. 4b). Intriguingly, when both H3K36me2 and H3K36me3 profiles are simultaneously shifted, IR showed a more significant shift compared to either H3K36me2 or H3K36me3 alone (Fig. 4c). As a control, unshifted H3K36me2/me3 exhibit no significant effect on IR switch (Fig. 4d). These results suggested that both H3K36me2 and H3K36me3 are involved in modulating IR shift, although a collaborative mechanism may exist in regulating IR.

However, the signals of H3K36 mono-, di-, and tri-methylation were also compared in differentially retained introns. Interestingly, both the levels of H3K36me2 and H3K36me3 at IRI-up introns were higher than those at IRI-down introns (Additional file 1: Figure S6), supporting the notion that H3K36me2/me3 probably demarcate IR in rice.

### Validation of the association between intron retention and H3K36me2 modification

**Fig. 5:**
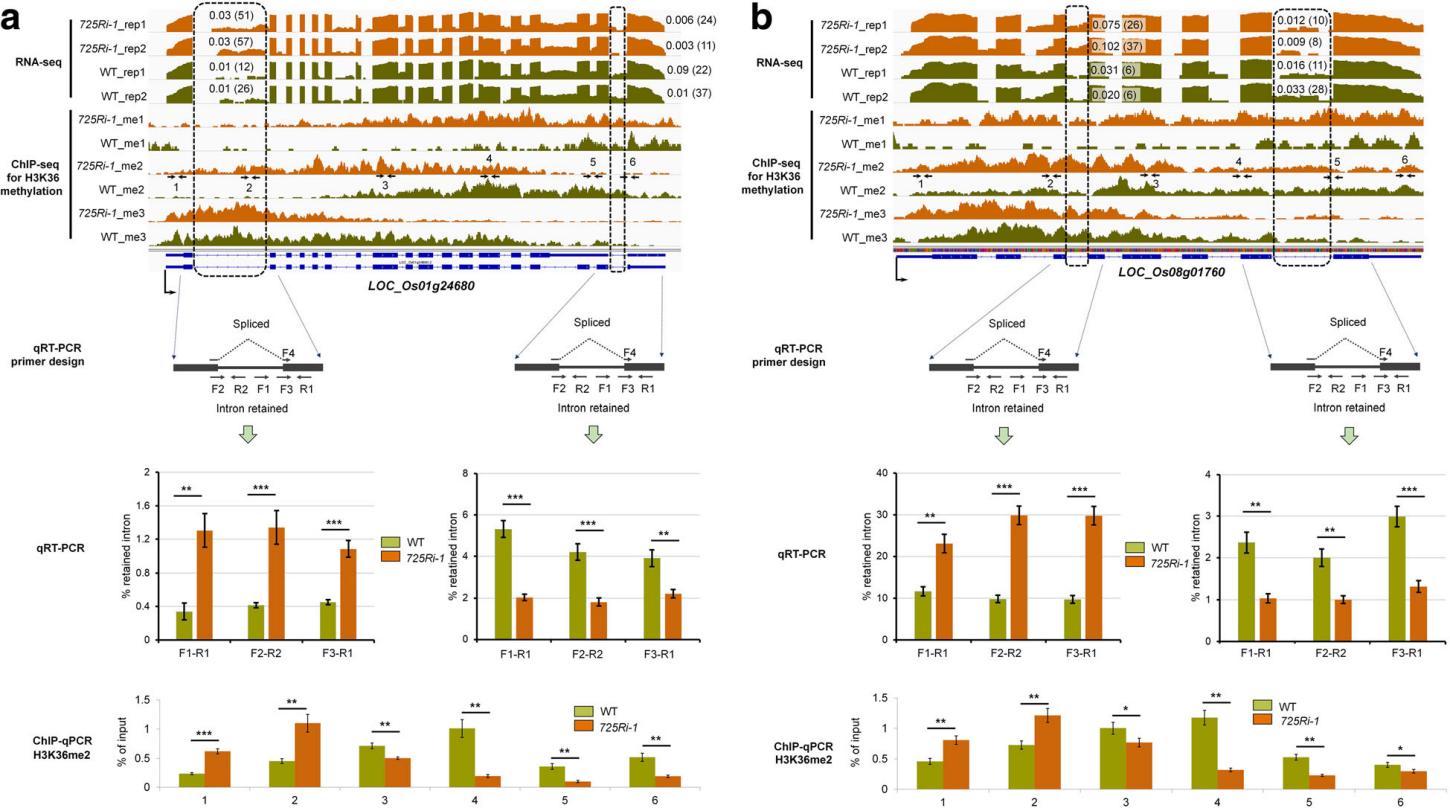
Validation of changes in IR events and associated alterations in H3K36me2 levels. **a**, **b** RNA-seq and ChIP-seq tracks, together with qRT-PCR and ChIP-PCR validations of two representative genes (**a** and **b**), which display higher levels of both IR and H3K36me2 at the 5′ end of gene body and lower levels at the 3′ end in *725Ri-1* mutant compared to WT rice plants. Two introns with IRI change validated by qRT-PCR are shown in *dashed rectangle*. IRI and read numbers (*in parentheses*) are shown near the corresponding introns. *Black arrow pairs* denote the positions of six primer sets that were used for ChIP-PCR validation for H3K36me2. Standard deviation (SD) of three qPCR replicates is shown. *, **, and *** stand for *p* value < 0.05, < 0.01 and < 0.001, respectively (*t* test)

The candidate gene approach was used to validate the potential link between H3K36me2 and IR. Two genes (*LOC_Os01g24680*, encodes putative 3-hydroxyacyl-CoA dehydrogenase; *LOC_Os08g01760*, encodes putative nutrition dehydrogenase) were selected from the 407 genes containing both IRI-up and IRI-down introns. For each gene, we selected six regions along the gene body to test H3K36me2 occupancy by ChIP-PCR. In addition, two IR events (one IRI-up and one IRI-down) for both genes were also examined by qRT-PCR in the same samples by multiple primer pairs spanning both intronic location and donor/acceptor sites (Fig. 5a, b**,** Additional file 2: Table S6). The results confirmed that the chromatin regions with increased levels of H3K36me2 modification produce increased IR events; accordingly, the chromatin regions with reduced H3K36me2 occupancy are associated with decreased IR events (Fig. 5).

### Transcripts with changed IR mainly accumulate in the nucleus

**Fig. 6:**
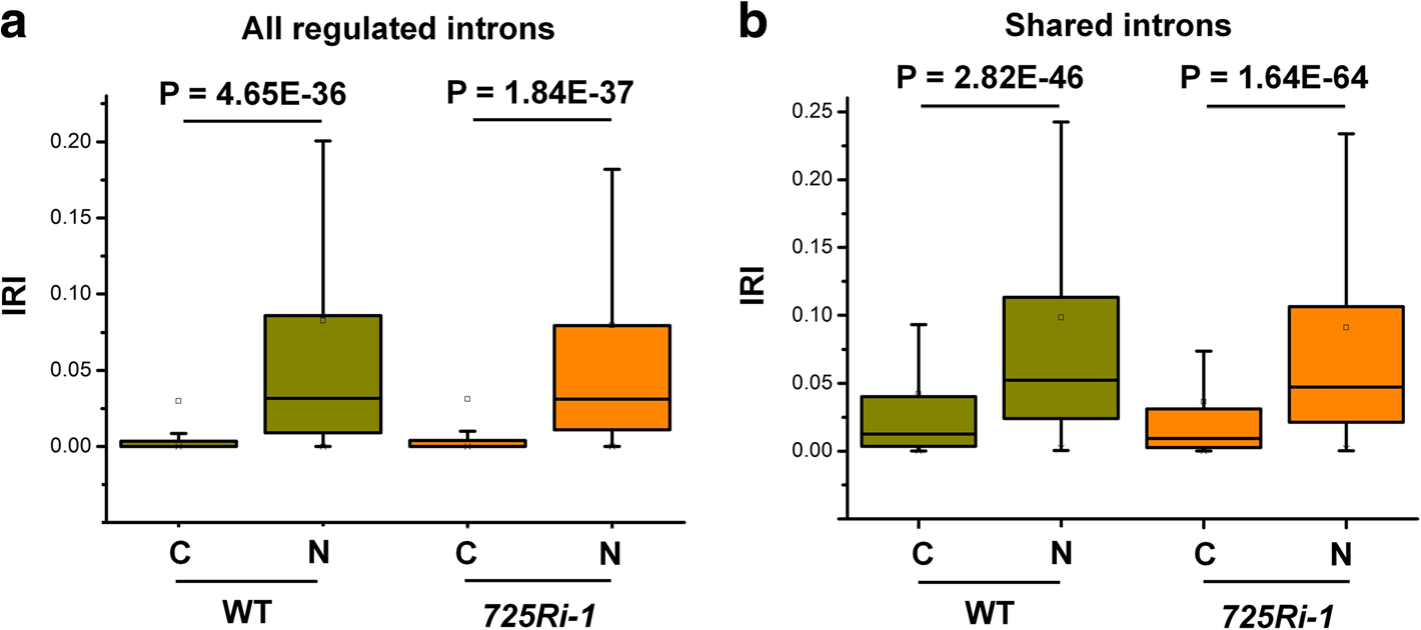
Box plot of IRI comparison between cytoplasmic (*C*) and nuclear (*N*) fractions in (**a**) all regulated introns (two-fold changes between *725Ri-1* and WT rice) and (**b**) retained introns shared between C and N. *IRI* intron retention index. *p* values of *t* test are shown

Cellular fractionation was performed to examine whether the IR transcripts are accumulated in the nucleus or cytoplasm. The success of cellular fractionation was confirmed by qRT-PCR of genes specifically expressed in the nucleus or cytoplasm (Additional file 1: Figure S7) [33, 34]. RNA-seq analysis on each fraction showed that transcripts with changed IR in rice dominantly accumulated in the nucleus (Fig. 6a, b**,** Additional file 2: Table S7). To further examine whether cytoplasmic NMD contributes to the transcript abundance, both *725Ri-1* and WT plants are treated with cycloheximide (CHX) to stabilize transcripts that are otherwise degraded by NMD, followed by RNA-seq analysis. Transcripts with a premature termination codon (PTC) showed an increased steady-state expression level upon CHX treatment (Additional file 1: Figure S8), indicating the successful inhibition of NMD. In contrast, genes with IR did not show an obvious expression increase upon CHX treatment (Additional file 1: Figure S9), suggesting cytoplasmic NMD has limited contribution to overall expression of IR loci in rice. These results indicate that intron-retained transcripts in rice are mainly accumulated in the nucleus rather than the cytoplasm and contribute to steady-state gene expression.

## Discussion

IR is thought to play a critical role in gene expression regulation in animals and plants. However, how IR is regulated in plants remains largely unknown. In this study we revealed that SDG725 may regulate global IR through H3K36me2/me3 modifications. We previously reported that SDG725 acts as an H3K36 methyltransferase and functions in promoting gene transcription [19, 20]. In both WT and *725Ri-1* plants, a positive correlation was observed between transcript abundance and H3K36me1/me2/me3 levels (Additional file 1: Figure S10a), extending previous findings obtained from other species [17, 35]. We discovered that changes in average H3K36me2/me3 occupancy level positively correlate with expression changes between *725Ri-1* with WT plants (Additional file 1: Figure S10b, c, left panels). The shift of H3K36me2/me3 affects IR but not expression level if the overall H3K36me2/me3 level does not change (Additional file 1: Figure S10b, c, right panels; Additional file 1: Figure S11). Taken together, we propose that SDG725 may control gene expression through two different mechanisms: (1) by modulating gene transcription via changing the overall levels of H3K36me2/me3, and (2) by regulating IR shift through H3K36me2/me3 shifts. How H3K36me2 or H3K36me2/me3 modulates IR in rice remains an open question. It might be achieved by reducing Pol II elongation rate and/or through a chromatin adaptor mechanism. In the first model, increased H3K36me2/me3 levels may slow down the elongation of Pol II. A longer dwell time of Pol II on introns may recruit splicing repressive factors or inhibit positive splicing factors to promote IR [2]. Alternatively, H3K36me2/me3 may be recognized by a specific “reader” protein, which interacts with splicing repressive factors to promote IR [4, 16]. Notably, the two models are not mutually exclusive and may act in concert to recruit splicing regulators. While significant expression change was not detected for known splicing factors between *725Ri-1* and WT rice (Additional file 2: Table S8), further investigations are warranted to identify H3K36me2/H3K36me3 reader(s) as well as downstream factors functionally involved in splicing regulation in rice.

The coupling of transcription and splicing is prevalent [36]. We observed a positive correlation between efficient splicing (or less IR) and gene expression level (Fig. 2b), indicating that IR negatively impacts the steady-state RNA level likely due to a higher degradation of IR transcripts. Moreover, increased IR events in the 5′ half of the gene in the *SDG725*-knockdown line suggest interaction between transcription and splicing machineries. Besides IR, other factors such as transcription activity are also possible contributors to steady-state mRNA expression.

To investigate the characteristics of retained introns, we examined several features including intron length, GC content, and splice site strength. Compared with spliced introns, retained introns tend to have much longer length, higher GC content, and weaker splice strength in the present rice study (Additional file 1: Figures S12–S14) [2, 3, 37]. Notably, the retained introns in animals tend to be shorter compared to spliced introns [2]. Because the average intron size is much bigger in animals (9519 bp in mouse and 11,538 bp in human) than in plants (407 bp for rice), we speculate that in animals a shorter intron is more likely to be recognized as an exon and has a higher tendency to be retained, whereas in plants a longer intron has a higher tendency to be recognized as an exon and is subsequently retained. The underlying mechanism is expected to be complicated and deserves further characterization.

The question of why the *SDG708* knockdown shows a global decrease of H3K36me2/me3 while the *SDG725* mutant displays a pattern shift is intriguing. One possible explanation is the different enzyme specificity between SDG725 and SDG708. Although both are H3K36 methyltransferases, SDG725 plays a major role in mono- to di- and di- to tri-methylation, while SDG708 functions more on me0 to mono-methylation according to our previous studies [19, 38]. As expected, knockdown of SDG708 reduced the level of H3K36me1 around the transcription start site (TSS) and transcription termination site (TTS) (Fig. 3b). The occupancy of H3K36me2 and H3K36me3 was also reduced (Fig. 3b), possibly due to the lack of H3K36me1. In addition, SDG725 but not SDG708 contains a CW domain, which can bind to H3K4 methylation that is enriched at promoter regions [39]. When SDG725 became limiting under the knockdown condition, it was preferentially recruited to the 5′ end of the gene, thereby showing more reductions of di- and tri-methylation of H3K36 at the 3′ portion of the gene body. This explains that the ChIP-seq result, represented as a relative distribution instead of an absolute level, showed a relative decrease of H3K36me2/me3 level in the 3′ half of the gene body but a relative increase of H3K36me2/me3 level close to the 5′ end (Fig. 3b).

Several lines of evidence support the notion that H3K36me2 in rice probably serves as the functional counterpart of H3K36me3 in animal cells [4, 16, 17]. H3K36me2 in plants and H3K36me3 in animals share similar occupancy profiles at transcribed regions (Fig. 3) [21]. The H3K36me3 patterns in both rice and Arabidopsis resemble the H3K4me3 or H3K79me3 profile in animals (Additional file 1: Figure S15) [40–43]. These observations suggest that plants and animals may employ distinct epigenetic factors (e.g., writers and reads) to coordinate transcription and post-transcriptional gene regulation, although the underlying regulatory principles are conserved during evolution. Our study demonstrated for the first time that a histone methyltransferase can regulate the position preference of IR, a unique phenomenon that deserves further molecular characterization.

## Conclusions

We found that depletion of the histone methyltransferase SDG725 in rice leads to position-specific alteration of intron retention (IR), a phenomenon that has not been demonstrated previously in any species. Further analyses support the model that H3K36me2/me3 but not H3K36me1 contribute to the IR alteration. As IR plays an important role in regulated gene expression of both plants and animals, the position-specific IR regulation revealed by this study further extends our knowledge regarding the complexity of gene regulatory networks.

## Methods

### Plant materials

Plants of *Oryza sativa* L. cv. Nipponbare were used in the present study. Seedlings used for RNA extraction and ChIP experiments were grown in artificial growth chambers under a long day (LD) photoperiod (14 h 30 °C: 10 h 28 °C, light: dark). For a rational association between ChIP-seq data and the RNA-seq data, the same set of plant samples was divided into two parts, one part for ChIP-seq library construction, and the other part for total RNA extraction followed by RNA-seq library preparation.

### Determination of H3K36 methylation level by mass spectrometry

The 14-day-old rice shoots were subjected to histone preparation based on an extraction method described previously [44]. Then, the prepared histones were separated according to molecular weight with sodium dodecyl sulfate-polyacrylamide gel electrophoresis (SDS-PAGE) using a standard procedure. The gels were excised at a certain molecular weight and derivatized by In-gel NHS and tested by mass spectrometry (MS) based on a method published recently [45].

### ChIP-seq and RNA-seq library construction and sequencing

We prepared ChIP-seq libraries using 14-day-old rice shoots with the published ChIP protocol [46]. Anti-monomethyl-H3K36 (ab9048), anti-dimethyl-H3K36 (ab9049), and anti-trimethyl-H3K36 (ab9050) were purchased from Abcam, Cambridge, UK. The resulting DNA and input control were subjected to ChIP-seq library construction as previously described [40]. For RNA-seq library construction, total RNA was extracted from 14-day-old rice shoots using TRIzol reagent (Invitrogen). PolyA+ RNA was enriched by selection with two rounds of Oligo(dT)25 Dynabeads (Invitrogen). A strand-specific RNA-seq library was constructed according to a dUTP-based protocol [47] adapted from Parkhomchuk et al. [48]. Both libraries were sequenced on an Illumina HiSeq2000 instrument to generate 50-bp single-end reads (for ChIP-seq) and 101-bp paired-end reads (for RNA-seq). All related ChIP-seq and RNA-seq data sets are summarized in Additional file 2: Table S9.

### Bioinformatics analysis of ChIP-seq data

The sequence tags for H3K36me1/2/3 were aligned against the complete reference genome of Nipponbare (japonica) rice (the MSU Rice Genome Annotation Project, Release 7 [49]) using Burrows-Wheeler Aligner (BWA) v0.6.1 [50], with the uniquely mapped reads then extracted with SAMtools [51]. To globally visualize the level of each histone modification along and around rice genes, genes longer than 500 bp were split into 300 blocks, with the upstream and downstream 2-kilobase (kb) regions each split into 100 blocks, respectively. The tag coverage of the aligned nonredundant ChIP-seq reads was then calculated for each block, normalized to 10 million reads, and calibrated to the input. A normalized tag intensity matrix was then obtained with custom scripts, and the average tag intensity was used to construct an aggregation plot. The normalized tag intensity of each gene feature, such as exons and introns, was also calculated as needed. For the heatmaps in Figs. 3 and 4, the *x*-axis denotes the relative position of a gene from 2 kb upstream of the TSS to 2 kb downstream of the TTS, and the *y*-axis denotes the normalized coverage of ChIP-seq reads calibrated by the input. The gene body (from TSS to TTS) was split into 300 bins, and the 2-kb upstream TSS and downstream TTS regions were split into 100 bins, respectively; thus, a total of 500 bins for each gene was used to calculate the coverage of ChIP-seq reads. To define the pattern shift of H3K36me2 or H3K36me3 in Fig. 4, we require that the front half (150 bins) of the gene body should have at least 30% of bins (45 bins) with a difference value (*725Ri-1* minus WT) greater than 0; at the same time, the latter half should have at least 30% of bins with a difference value smaller than 0. The rest are then considered as not having a pattern shift.

### Bioinformatics analysis of RNA-seq data

To calculate the expression abundance of transcripts derived from each gene, we used a series of three programs: Bowtie v1.0.0 [52], TopHat2 v2.09 [53], and Cufflinks v2.1.1 [54, 55]. Briefly, the adaptor-removed raw RNA-seq reads were first aligned to Nipponbare (japonica) rice annotated transcripts from Release 7 of the MSU Rice Genome Annotation Project [49] with Bowtie to estimate insert fragment sizes and standard deviations, which were in turn used as parameter values in TopHat2. TopHat2 was then used to align the paired-end reads to the complete reference genome as mentioned above. Quantification of transcripts and genes, normalized for gene length, was performed with Cufflinks, as represented by fragments per kilobase of exon per million fragments uniquely mapped (FPKM). A differential expression analysis was performed using Cuffdiff, a subpackage of Cufflinks. A cutoff of at least twofold change, FPKM greater than 1 in at least one sample in a sample pair, and a *p* value smaller than 0.01 was used as the threshold to define DEGs.

### Determination of changes in intron retention

To determine the IR and minimize the interference from exons, a new set of introns was acquired so that only introns or intron fragments that do not overlap with any exons were used as corresponding actual introns. In a similar way, we get a new set of exons that do not overlap with any other introns. To determine the IR events, the two exons neighboring an intron must be expressed (i.e., each with an FPKM value greater than 1), and there should be at least three reads supporting the IR events. To evaluate the IR level for a given intron, we introduced the intron retention index (IRI) for an individual intron to quantify its IR level. We obtained the IRI in the following way. The IR level (determined by FPKM) for a given intron was divided by the mean value of the expression level of its neighboring exons (determined by FPKM). To evaluate the IR changes between *725Ri-1* mutants and WT plants, the IRI ratio between the two samples was used, and those exceeding a twofold change were considered as differentially changed (up-regulated or down-regulated) IR events. To compare the location distribution difference between mutant and WT rice, we applied the two-sample Kolmogorov-Smirnov test, which is one of the most useful and general nonparametric statistical methods for comparing two samples without replication, as it is sensitive to differences in both location and shape of the empirical cumulative distribution functions of the two samples [56].

### qRT-PCR for validation of intron retention

Total RNA was extracted from 14-day-old rice shoots using TRIzol reagent (Invitrogen). Reverse transcription was performed using Improm-II Reverse Transcriptase (Promega, Madison, WI, USA) according to the standard protocol provided. Quantitative PCR was performed using the gene-specific primers listed in Additional file 2: Tables S3 and S6.

### ChIP-PCR for validation

The 14-day-old rice shoots were used in ChIP-PCR assays as previously described [46]. Anti-dimethyl-H3K36 (ab9049) was purchased from Abcam. ChIP assays were performed according to a previous method [19]. Quantitative PCR was performed to determine the enrichment of immunoprecipitated DNA using a kit from Takara, Otsu, Japan. The gene-specific primers are listed in Additional file 2: Table S6.

### Nuclear/cytoplasmic fractionation extraction and quality assay

To determine whether intron-retained transcripts accumulate in the nucleus or cytoplasm, nuclear/cytoplasmic fractionation was performed according to a published method [33]. As quality controls for the fractionation, we evaluated the relative expression abundance of a cytoplasmic marker (*Actin1*) and nuclear markers (*Pri-miR156d*, *Pri-miR156h*, and *Pri-miR156b*) in the isolated fractions [33, 34]. Strand-specific RNA-seq libraries were constructed using both nuclear and cytoplasmic RNA according to a dUTP-based protocol [47] adapted from Parkhomchuk et al. [48].

### Cycloheximide treatment and prediction of potential targets of nonsense-mediated mRNA decay

To evaluate the extent that NMD may be involved in the metabolism of intron-retained transcripts, we treated both *725Ri-1* and WT 2-week-old rice plants with cycloheximide (CHX) (which can block the translation process and rescue the NMD-targeted transcripts that would otherwise be degraded [57]) according to a published method with minor modifications [58, 59]. The same batch of dimethylsulfoxide (DMSO) treatments was used as the control. Then the treated rice samples were used for RNA extraction, followed by RNA-seq library preparation and sequencing as described above. After quality control, the raw sequenced reads were aligned to the rice reference genome as described above, then StringTie was used to assemble transcripts for each sample and combine them together with Cuffcompare, a subpackage of Cufflinks [55]. Then the annotated unique start codon located on the assembled transcript was used for the open reading frame (ORF) prediction. A stop codon was defined as a premature termination codon (PTC) if it was located more than 50 nucleotides upstream of the last exon-exon junction, which is a well-known feature of NMD targets [60]. Lastly, a PTC-containing transcript was defined as a potential NMD target if its accumulative abundance increased more than twofold in *725Ri-1* rice compared to WT plants, with a *p* value smaller than 0.05, determined by Cuffdiff [55].

### Analysis of splice site strength

MaxEntScan [37] was used to calculate maximum entropy scores for 9 bp spanning the 5′ (donor) splice sites (3 bp in the exon and 6 bp in the intron) and 23 bp spanning the 3′ (acceptor) splice sites (20 bp in the intron and 3 bp in the exon), respectively.

## Additional files

Additional file 1: **Figure S1.** The quantification of H3K36 methylation in *725Ri-1.* and WT plants. **Figure S2.** Differential gene expression between *725Ri-1.* and WT rice. **Figure S3.** Accumulative plot of up-regulated (*725Ri-1.*_IRI_up) and down-regulated (*725Ri-1.*_IRI_down) IR events with different intron length coverage. **Figure S4.** Distribution of up-regulated (*red.*) and down-regulated (*blue.*) intron retention (IR) events between *725Ri-1.* and wild-type (WT) rice (a) and between *708Ri-1.* and WT (b). **Figure S5.** Schematic diagram illustrating the way to transform the H3K36 methylation changes between the *725Ri-1.* and wild-type plants at individual gene level. **Figure S6.** Box plot for levels of H3K36 methylations at IRI-up or IRI-down introns. **Figure S7.** qPCR validation of quality of cellular fractionation in wild-type rice. **Figure S8.** Box plot for expression level of transcripts with premature termination codon. **Figure S9.** Box plot of gene expression before and after cycloheximide (CHX) treatment in either wild-type or *725Ri-1.* rice. **Figure S10.** H3K36 methylations associate with gene expression levels. **Figure S11.** Distribution of up-regulated (*red.*) and down-regulated (*blue.*) intron retention (IR) events in genes with no obvious changes (≤ 2-fold) in total levels of H3K36me2 (a) or H3K36me3 (b). **Figure S12.** Box plot for intron length in two groups of introns by distinct methods in calculating the degree of intron retention. **Figure S13.** Box plot for GC percentage in two groups of introns. **Figure S14.** Box plot for maximum entropy score of 5′ splice site (a) and 3′ splice site (b) in two groups of introns. **Figure S15.** Comparison of H3K36me2/me3 distribution across gene body between rice (a) and Arabidopsis (b). (DOCX 2596 kb)

Additional file 2: **Table S1.** Summary of the RNA-seq libraries. **Table S2.** Alternative splicing events discovered between rice RNA-seq data sets in this study compared with annotation by using SplAdder. **Table S3.** Primers used for validating intron-retention events. **Table S4.** The numbers of IRI changed introns and genes. **Table S5.** Summary of the ChIP-seq libraries. **Table S6.** Primers used to validate intron retention in the same gene by qRT-PCR and those used to validate H3K36me2 by ChIP-PCR. **Table S7.** Number of retained introns for previously identified IRI changed introns in nucleus or cytoplasm. **Table S8.** Expression changes of annotated rice splicing factors in our data. **Table S9.** Detailed information for ChIP-seq and RNA-seq data sets. (DOCX 43 kb)

## References

[1] Wong JJ, Ritchie W, Ebner OA, Selbach M, Wong JW, Huang Y, Gao D, Pinello N, Gonzalez M, Baidya K (2013). Orchestrated intron retention regulates normal granulocyte differentiation. Cell..

[2] Braunschweig U, Barbosa-Morais NL, Pan Q, Nachman EN, Alipanahi B, Gonatopoulos-Pournatzis T, Frey B, Irimia M, Blencowe BJ (2014). Widespread intron retention in mammals functionally tunes transcriptomes. Genome Res..

[3] Ni T, Yang W, Han M, Zhang Y, Shen T, Nie H, Zhou Z, Dai Y, Yang Y, Liu P (2016). Global intron retention mediated gene regulation during CD4+ T cell activation. Nucleic Acids Res..

[4] Guo R, Zheng L, Park JW, Lv R, Chen H, Jiao F, Xu W, Mu S, Wen H, Qiu J (2014). BS69/ZMYND11 reads and connects histone H3.3 lysine 36 trimethylation-decorated chromatin to regulated pre-mRNA processing. Mol Cell..

[5] Shahzad K, Rauf M, Ahmed M, Malik ZA, Habib I, Ahmed Z, Mahmood K, Ali R, Masmoudi K, Lemtiri-Chlieh F (2015). Functional characterisation of an intron retaining K(+) transporter of barley reveals intron-mediated alternate splicing. Plant Biol..

[6] Remy E, Cabrito TR, Batista RA, Hussein MA, Teixeira MC, Athanasiadis A, Sa-Correia I, Duque P (2014). Intron retention in the 5'UTR of the novel ZIF2 transporter enhances translation to promote zinc tolerance in Arabidopsis. PLoS Genet..

[7] Barbazuk WB, Fu Y, McGinnis KM (2008). Genome-wide analyses of alternative splicing in plants: opportunities and challenges. Genome Res..

[8] Ner-Gaon H, Halachmi R, Savaldi-Goldstein S, Rubin E, Ophir R, Fluhr R (2004). Intron retention is a major phenomenon in alternative splicing in Arabidopsis. Plant J.

[9] Min XJ, Powell B, Braessler J, Meinken J, Yu F, Sablok G (2015). Genome-wide cataloging and analysis of alternatively spliced genes in cereal crops. BMC Genomics..

[10] Wang BB, Brendel V (2006). Genomewide comparative analysis of alternative splicing in plants. Proc Natl Acad Sci U S A..

[11] Panahi B, Mohammadi SA, Ebrahimi Khaksefidi R, Fallah Mehrabadi J, Ebrahimie E (2015). Genome-wide analysis of alternative splicing events in Hordeum vulgare: highlighting retention of intron-based splicing and its possible function through network analysis. FEBS Lett..

[12] Li Y, Dai C, Hu C, Liu Z, Kang C (2017). Global identification of alternative splicing via comparative analysis of SMRT- and Illumina-based RNA-seq in strawberry. Plant J.

[13] Gohring J, Jacak J, Barta A (2014). Imaging of endogenous messenger RNA splice variants in living cells reveals nuclear retention of transcripts inaccessible to nonsense-mediated decay in Arabidopsis. Plant Cell..

[14] Boothby TC, Zipper RS, van der Weele CM, Wolniak SM (2013). Removal of retained introns regulates translation in the rapidly developing gametophyte of Marsilea vestita. Dev Cell..

[15] Luco RF, Allo M, Schor IE, Kornblihtt AR, Misteli T (2011). Epigenetics in alternative pre-mRNA splicing. Cell..

[16] Luco RF, Pan Q, Tominaga K, Blencowe BJ, Pereira-Smith OM, Misteli T (2010). Regulation of alternative splicing by histone modifications. Science..

[17] Kolasinska-Zwierz P, Down T, Latorre I, Liu T, Liu XS, Ahringer J (2009). Differential chromatin marking of introns and expressed exons by H3K36me3. Nat Genet..

[18] Pajoro A, Severing E, Angenent GC, Immink RGH (2017). Histone H3 lysine 36 methylation affects temperature-induced alternative splicing and flowering in plants. Genome Biol..

[19] Sui P, Jin J, Ye S, Mu C, Gao J, Feng H, Shen WH, Yu Y, Dong A (2012). H3K36 methylation is critical for brassinosteroid-regulated plant growth and development in rice. Plant J.

[20] Sui PF, Shi JL, Gao XY, Shen WH, Dong AW (2013). H3K36 methylation is involved in promoting rice flowering. Mol Plant..

[21] Liu B, Wei G, Shi J, Jin J, Shen T, Ni T, Shen WH, Yu Y, Dong A. SET DOMAIN GROUP 708. A histone H3 lysine 36-specific methyltransferase, controls flowering time in rice (Oryza sativa). New Phytol 2015. 10.1111/nph.13768. 10.1111/nph.13768 26639303

[22] Kahles A, Ong CS, Zhong Y, Ratsch G (2016). SplAdder: identification, quantification and testing of alternative splicing events from RNA-Seq data. Bioinformatics..

[23] Chamala S, Feng G, Chavarro C, Barbazuk WB (2015). Genome-wide identification of evolutionarily conserved alternative splicing events in flowering plants. Front Bioeng Biotechnol..

[24] Yap K, Lim ZQ, Khandelia P, Friedman B, Makeyev EV (2012). Coordinated regulation of neuronal mRNA steady-state levels through developmentally controlled intron retention. Genes Dev..

[25] Kalyna M, Simpson CG, Syed NH, Lewandowska D, Marquez Y, Kusenda B, Marshall J, Fuller J, Cardle L, McNicol J (2012). Alternative splicing and nonsense-mediated decay modulate expression of important regulatory genes in Arabidopsis. Nucleic Acids Res..

[26] Marquez Y, Brown JW, Simpson C, Barta A, Kalyna M (2012). Transcriptome survey reveals increased complexity of the alternative splicing landscape in Arabidopsis. Genome Res..

[27] Marquez Y, Hopfler M, Ayatollahi Z, Barta A, Kalyna M (2015). Unmasking alternative splicing inside protein-coding exons defines exitrons and their role in proteome plasticity. Genome Res..

[28] Napoli N, Ghelli R, Brunetti P, De Paolis A, Cecchetti V, Tsuge T, Serino G, Matsui M, Mele G, Rinaldi G et al. A newly identified flower-specific splice variant of AUXIN RESPONSE FACTOR8 regulates stamen elongation and endothecium lignification in Arabidopsis. Plant Cell. 2018. 10.1105/tpc.17.00840. 10.1105/tpc.17.00840 PMC5894849 29514943

[29] Jacob AG, Smith CWJ (2017). Intron retention as a component of regulated gene expression programs. Hum Genet..

[30] Tahmasebi S, Jafarnejad SM, Tam IS, Gonatopoulos-Pournatzis T, Matta-Camacho E, Tsukumo Y, Yanagiya A, Li W, Atlasi Y, Caron M (2016). Control of embryonic stem cell self-renewal and differentiation via coordinated alternative splicing and translation of YY2. Proc Natl Acad Sci U S A..

[31] Thiele A, Nagamine Y, Hauschildt S, Clevers H (2006). AU-rich elements and alternative splicing in the beta-catenin 3'UTR can influence the human beta-catenin mRNA stability. Exp Cell Res..

[32] Sun S, Zhang Z, Sinha R, Karni R, Krainer AR (2010). SF2/ASF autoregulation involves multiple layers of post-transcriptional and translational control. Nat Struct Mol Biol..

[33] Wang W, Ye R, Xin Y, Fang X, Li C, Shi H, Zhou X, Qi Y (2011). An importin beta protein negatively regulates MicroRNA activity in Arabidopsis. Plant Cell..

[34] Ye R, Wang W, Iki T, Liu C, Wu Y, Ishikawa M, Zhou X, Qi Y (2012). Cytoplasmic assembly and selective nuclear import of Arabidopsis Argonaute4/siRNA complexes. Mol Cell..

[35] Hon G, Wang W, Ren B (2009). Discovery and annotation of functional chromatin signatures in the human genome. PLoS Comput Biol..

[36] Tilgner H, Knowles DG, Johnson R, Davis CA, Chakrabortty S, Djebali S, Curado J, Snyder M, Gingeras TR, Guigo R (2012). Deep sequencing of subcellular RNA fractions shows splicing to be predominantly co-transcriptional in the human genome but inefficient for lncRNAs. Genome Res..

[37] Yeo G, Burge CB (2004). Maximum entropy modeling of short sequence motifs with applications to RNA splicing signals. J Comput Biol..

[38] Liu B, Wei G, Shi J, Jin J, Shen T, Ni T, Shen WH, Yu Y, Dong A (2016). SET DOMAIN GROUP 708, a histone H3 lysine 36-specific methyltransferase, controls flowering time in rice (Oryza sativa). New Phytol..

[39] Hoppmann V, Thorstensen T, Kristiansen PE, Veiseth SV, Rahman MA, Finne K, Aalen RB, Aasland R (2011). The CW domain, a new histone recognition module in chromatin proteins. EMBO J..

[40] Barski A, Cuddapah S, Cui K, Roh TY, Schones DE, Wang Z, Wei G, Chepelev I, Zhao K (2007). High-resolution profiling of histone methylations in the human genome. Cell..

[41] Wang Z, Zang C, Rosenfeld JA, Schones DE, Barski A, Cuddapah S, Cui K, Roh TY, Peng W, Zhang MQ (2008). Combinatorial patterns of histone acetylations and methylations in the human genome. Nat Genet..

[42] Kuntimaddi A, Achille NJ, Thorpe J, Lokken AA, Singh R, Hemenway CS, Adli M, Zeleznik-Le NJ, Bushweller JH (2015). Degree of recruitment of DOT1L to MLL-AF9 defines level of H3K79 di- and tri-methylation on target genes and transformation potential. Cell Rep..

[43] Li Y, Mukherjee I, Thum KE, Tanurdzic M, Katari MS, Obertello M, Edwards MB, McCombie WR, Martienssen RA, Coruzzi GM (2015). The histone methyltransferase SDG8 mediates the epigenetic modification of light and carbon responsive genes in plants. Genome Biol..

[44] Yu Y, Dong A, Shen WH (2004). Molecular characterization of the tobacco SET domain protein NtSET1 unravels its role in histone methylation, chromatin binding, and segregation. Plant J..

[45] Chen J, Gao J, Peng M, Wang Y, Yu Y, Yang P, Jin H (2015). In-gel NHS-propionate derivatization for histone post-translational modifications analysis in Arabidopsis thaliana. Anal Chim Acta..

[46] Ding B, Zhu Y, Bu ZY, Shen WH, Yu Y, Dong AW. SDG714 regulates specific gene expression and consequently affects plant growth via H3K9 dimethylation. J Integr Plant Biol. 2010;52(4):420-30. 10.1111/j.1744-7909.2010.00927.x. 10.1111/j.1744-7909.2010.00927.x 20377704

[47] Zhong S, Joung JG, Zheng Y, Chen YR, Liu B, Shao Y, Xiang JZ, Fei Z, Giovannoni JJ (2011). High-throughput Illumina strand-specific RNA sequencing library preparation. Cold Spring Harb Protoc..

[48] Parkhomchuk D, Borodina T, Amstislavskiy V, Banaru M, Hallen L, Krobitsch S, Lehrach H, Soldatov A (2009). Transcriptome analysis by strand-specific sequencing of complementary DNA. Nucleic Acids Res..

[49] Kawahara Y, de la Bastide M, Hamilton JP, Kanamori H, McCombie WR, Ouyang S, Schwartz DC, Tanaka T, Wu J, Zhou S (2013). Improvement of the Oryza sativa Nipponbare reference genome using next generation sequence and optical map data. Rice.

[50] Li H, Durbin R (2009). Fast and accurate short read alignment with Burrows-Wheeler transform. Bioinformatics..

[51] Li H, Handsaker B, Wysoker A, Fennell T, Ruan J, Homer N, Marth G, Abecasis G, Durbin R, Genome Project Data Processing S (2009). The Sequence Alignment/Map format and SAMtools. Bioinformatics..

[52] Langmead B, Trapnell C, Pop M, Salzberg SL (2009). Ultrafast and memory-efficient alignment of short DNA sequences to the human genome. Genome Biol..

[53] Kim D, Pertea G, Trapnell C, Pimentel H, Kelley R, Salzberg SL (2013). TopHat2: accurate alignment of transcriptomes in the presence of insertions, deletions and gene fusions. Genome Biol..

[54] Roberts A, Trapnell C, Donaghey J, Rinn JL, Pachter L (2011). Improving RNA-Seq expression estimates by correcting for fragment bias. Genome Biol..

[55] Trapnell C, Williams BA, Pertea G, Mortazavi A, Kwan G, van Baren MJ, Salzberg SL, Wold BJ, Pachter L (2010). Transcript assembly and quantification by RNA-Seq reveals unannotated transcripts and isoform switching during cell differentiation. Nat Biotechnol..

[56] Arnold TB, Emerson JW (2011). Nonparametric goodness-of-fit tests for discrete null istributions. R J.

[57] Carter MS, Doskow J, Morris P, Li S, Nhim RP, Sandstedt S, Wilkinson MF (1995). A regulatory mechanism that detects premature nonsense codons in T-cell receptor transcripts in vivo is reversed by protein synthesis inhibitors in vitro. J Biol Chem..

[58] Jeong HJ, Kim YJ, Kim SH, Kim YH, Lee IJ, Kim YK, Shin JS (2011). Nonsense-mediated mRNA decay factors, UPF1 and UPF3, contribute to plant defense. Plant Cell Physiol..

[59] Shen Y, Wu X, Liu D, Song S, Liu D, Wang H (2016). Cold-dependent alternative splicing of a Jumonji C domain-containing gene MtJMJC5 in Medicago truncatula. Biochem Biophys Res Commun..

[60] Maquat LE (2002). Nonsense-mediated mRNA decay. Curr Biol..

