# Supplementary material for: Position-specific intron retention is mediated by the histone methyltransferase SDG725
Source: BMC Biol. 2018 Apr 30;16:44. doi: 10.1186/s12915-018-0513-8 (PMC5925840; doi:10.1186/s12915-018-0513-8)
Supplement: Supplementary file 1 — Figure S1. The quantification of H3K36 methylation in 725Ri-1. and WT plants. Figure S2. Differential gene expression between 725Ri-1. and WT rice. Figure S3. Accumulative plot of up-regulated (725Ri-1._IRI_up) and down-regulated (725Ri-1._IRI_down) IR events with different intron length coverage. Figure S4. Distribution of up-regulated (red.) and down-regulated (blue.) intron retention (IR) events between 725Ri-1. and wild-type (WT) rice (a) and between 708Ri-1. and WT (b). Figure S5. Schematic diagram illustrating the way to transform the H3K36 methylation changes between the 725Ri-1. and wild-type plants at individual gene level. Figure S6. Box plot for levels of H3K36 methylations at IRI-up or IRI-down introns. Figure S7. qPCR validation of quality of cellular fractionation in wild-type rice. Figure S8. Box plot for expression level of transcripts with premature termination codon. Figure S9. Box plot of gene expression before and after cycloheximide (CHX) treatment in either wild-type or 725Ri-1. rice. Figure S10. H3K36 methylations associate with gene expression levels. Figure S11. Distribution of up-regulated (red.) and down-regulated (blue.) intron retention (IR) events in genes with no obvious changes (≤ 2-fold) in total levels of H3K36me2 (a) or H3K36me3 (b). Figure S12. Box plot for intron length in two groups of introns by distinct methods in calculating the degree of intron retention. Figure S13. Box plot for GC percentage in two groups of introns. Figure S14. Box plot for maximum entropy score of 5′ splice site (a) and 3′ splice site (b) in two groups of introns. Figure S15. Comparison of H3K36me2/me3 distribution across gene body between rice (a) and Arabidopsis (b). (DOCX 2596 kb) [file 12915_2018_513_MOESM1_ESM.docx]

**Position-specific intron retention is mediated by the histone methyltransferase SDG725**

Gang Wei, Kunpeng Liu, Ting Shen, Jinlei Shi, Bing Liu, Wenjing Yang, Miao Han, Maolin Peng, Haihui Fu, Yifan Song, Jun Zhu, Aiwu Dong, Ting Ni

**Supporting Figures**

**Figure S1** - The quantification of H3K36 methylation in *725Ri-1* and WT plants.

**Figure S2** - Differential gene expression between *725Ri-1* and WT rice.

**Figure S3** - Accumulative plot of up-regulated (*725Ri-1*_IRI_up) and down-regulated (*725Ri-1*_IRI_down) IR events with different intron length coverage.

**Figure S4** - Distribution of up-regulated (red) and down-regulated (blue) intron retention (IR) events between *725Ri-1* and wild-type (WT) rice (a), and between *708Ri-1* and WT (b).

**Figure S5** - The schematic diagram illustrating the way to transform the H3K36 methylation changes between the *725Ri-1* and the wild-type plants at individual gene level.

**Figure S6** - Box plots for levels of H3K36 methylations at IRI-up or IRI-down introns.

**Figure S7** - qPCR validation of quality of cellular fractionation in wild type rice.

**Figure S8** - Box plot for expression level of transcripts with premature termination codon.

**Figure S9** - Box plot of gene expression before and after cycloheximide (CHX) treatment in either wild-type or 725Ri-1 rice.

**Figure S10** - H3K36 methylations associate with gene expression levels.

**Figure S11** - Distribution of up-regulated (red) and down-regulated (blue) intron retention (IR) events in genes with no obvious changes (≤ 2-fold) in total levels of H3K36me2 (a) or H3K36me3 (b).

**Figure S12** - Box plot for intron length in two groups of introns by distinct methods in calculating the degree of intron retention.

**Figure S13** - Box plot for GC percentage in two groups of introns.

**Figure S14** - Box plot for maximum entropy score of 5′ splice site (a) and 3′ splice site (b) in two groups of introns.

**Figure S15** - Comparison of H3K36me2/3 distribution across gene body between rice (a) and Arabidopsis (b).


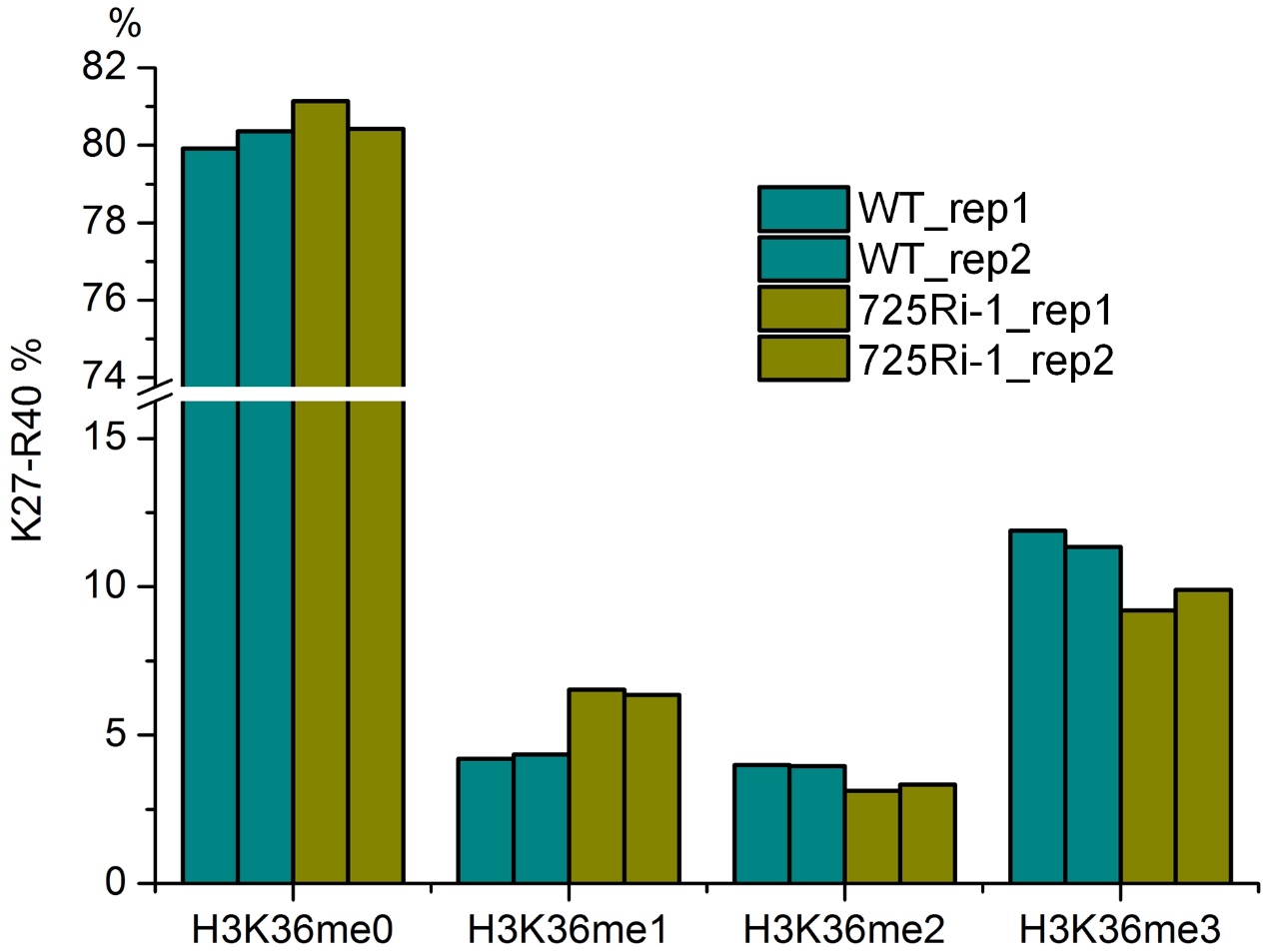


**Figure S1 - The quantification of H3K36 methylation in *725Ri-1* and WT plants.**

The overall levels of H3K36me0, H3K36me1, H3K36me2 and H3K36me3 in the *725Ri-1* mutant and wild-type (WT) rice plants were calculated by summing the specific peptides by mass spectrometry. Y axis denotes the percentage of K36 modification [45]. Rep1 and rep2 represent two biological replicates.


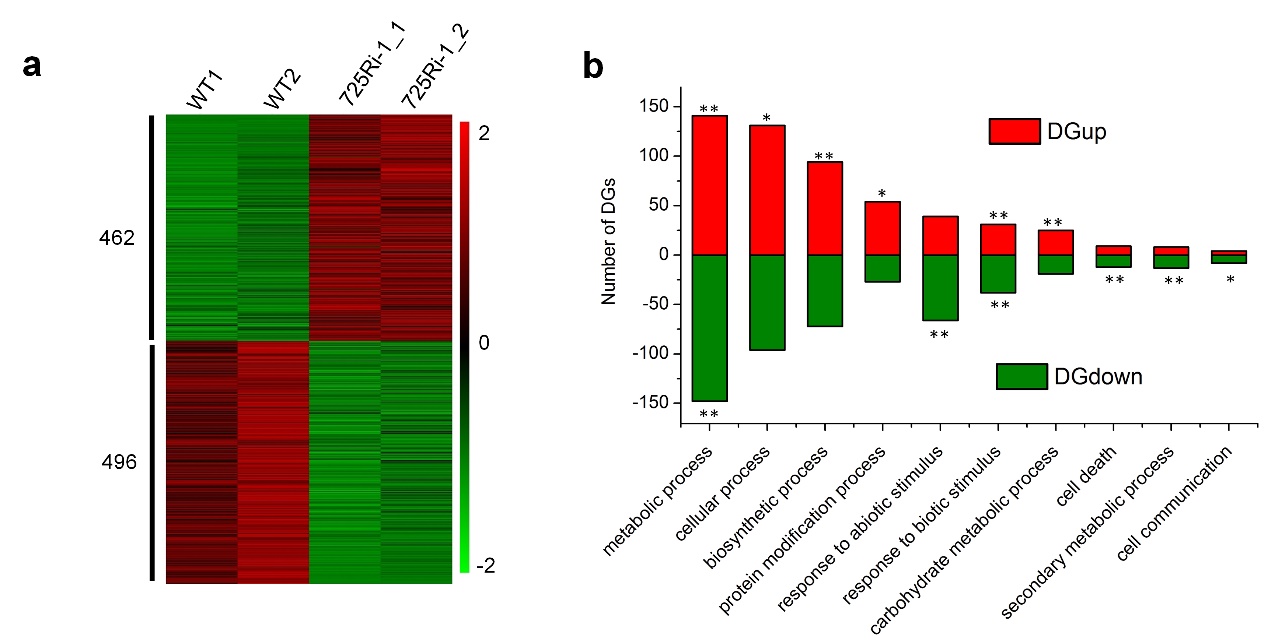


**Figure S2 - Differential gene expression between *725Ri-1* and WT rice.**

**(a)** Heatmap of differentially expressed genes (DGs). For each sample, Z-score transformed expression values of two biological replicates were used to draw the heat map (see the Methods). The numbers of up-regulated and down-regulated genes are noted on the left, respectively. Value of Z score is illustrated on the right by color bar. **(b)** Gene Ontology analysis for DGs. GO_BP was used for the analysis, with enrichment (p < 0.05) for DGup or DGdown genes, * and ** denote p<0.05 and p<0.01, respectively.


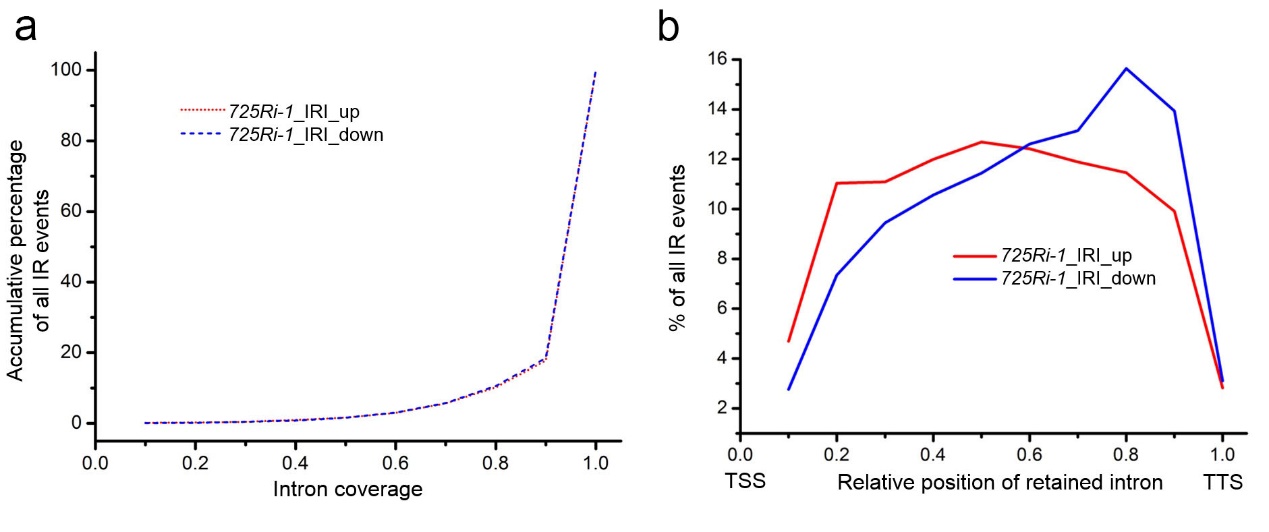


**Figure S3 -** **Accumulative plot of up-regulated (*725Ri-1*_IRI_up) and down-regulated (*725Ri-1*_IRI_down) IR events with different intron length coverage.**

Most of the IR events have a coverage greater than 80%.


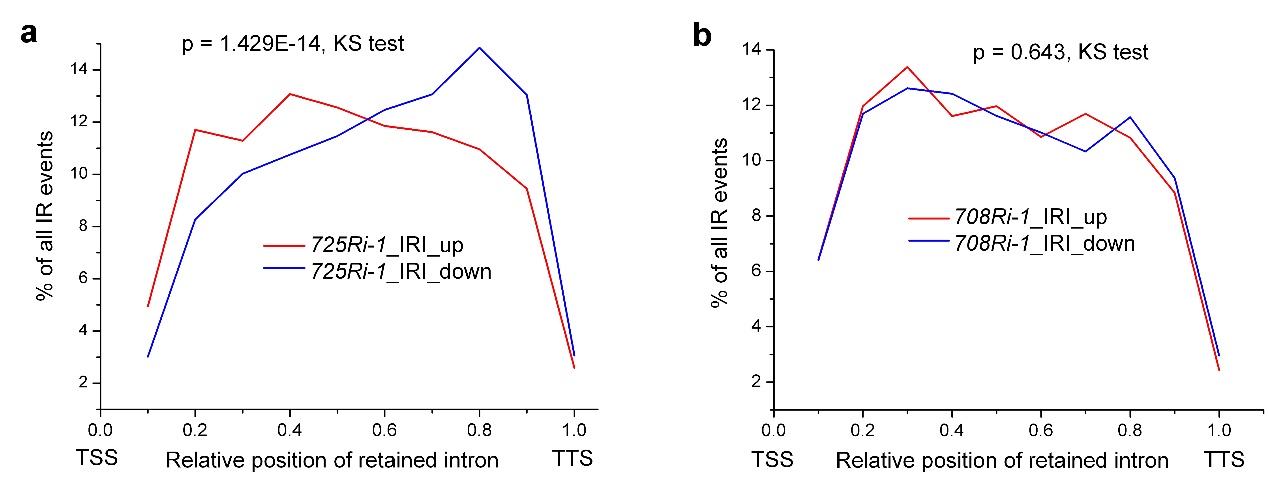


**Figure S4 - Distribution of up-regulated (red) and down-regulated (blue) intron retention (IR) events between *725Ri-1* and wild-type (WT) rice (a), and between *708Ri-1* and WT (b).**

A two-fold change in IRI was applied as a cutoff to define differential IR events. The retained introns need to be supported by at least 3 reads and host gene expression ≥ 1 FPKM. Average IRI value of two biological replicates of both wild-type and mutant was used for location distribution KS test. TSS and TTS stands for transcription start site and transcription termination site, respectively.


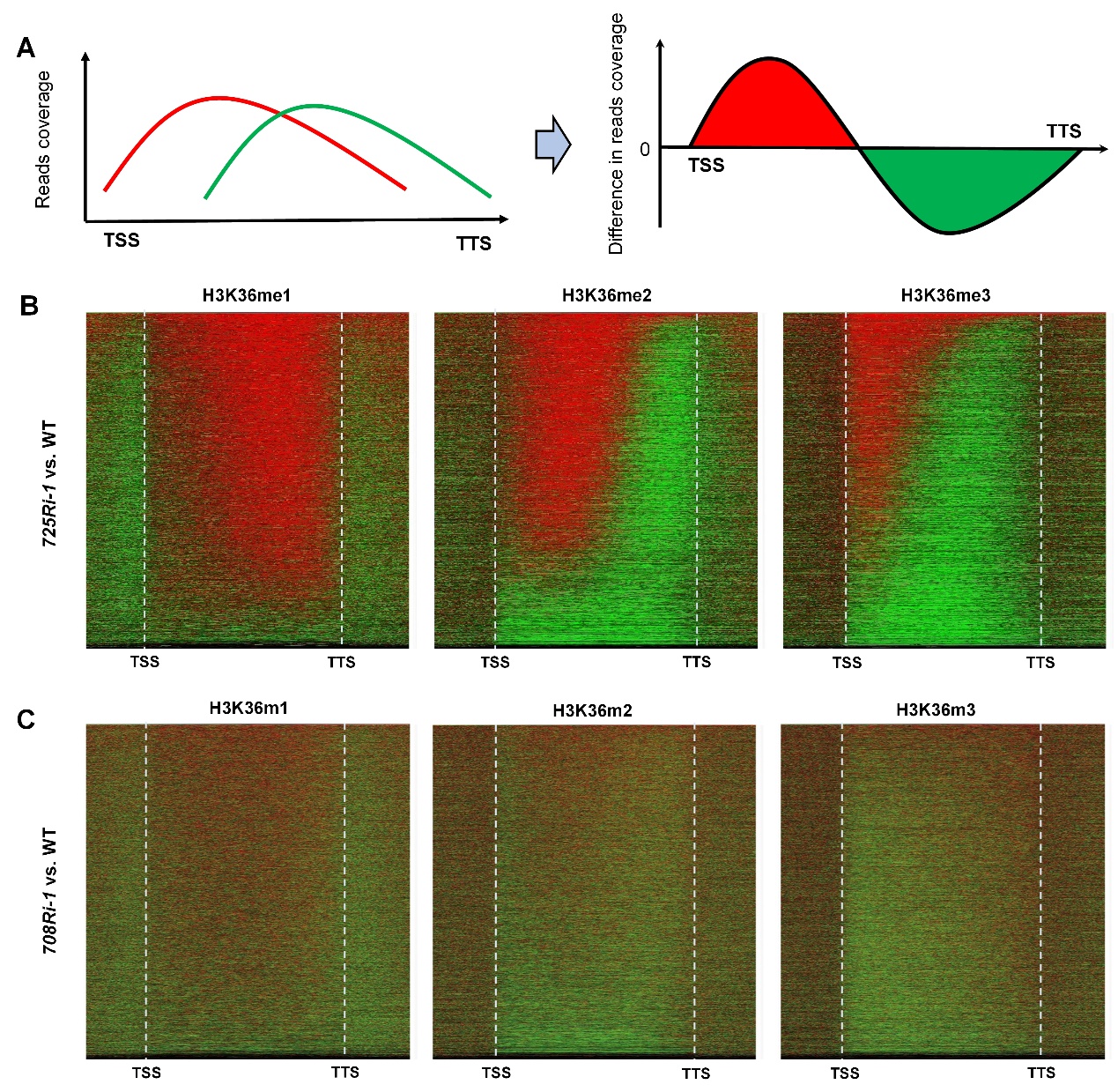


**Figure S5 - The schematic diagram illustrating the way to transform the H3K36 methylation changes between the *725Ri-1* and the wild-type plants at individual gene level.**

For a relative position of a gene (x axis), there are two coverage values (y1, y2) for ChIP-seq reads for mutant (y1, red) and WT (y2, green), respectively. The difference between the two values (Δy = y1 - y2) was used to demonstrate the status changes of H3K36 methylation. Genes with pattern shift would show a pattern similar to sine distribution. Using this method, one can distinguish the H3K36 methylation changes for each gene after knockdown (KD) of *SDG725* based on red (ChIP-seq signal increased in *725Ri-1* compared to WT) or green (ChIP-seq signal decreased in *725Ri-1*) heatmap.


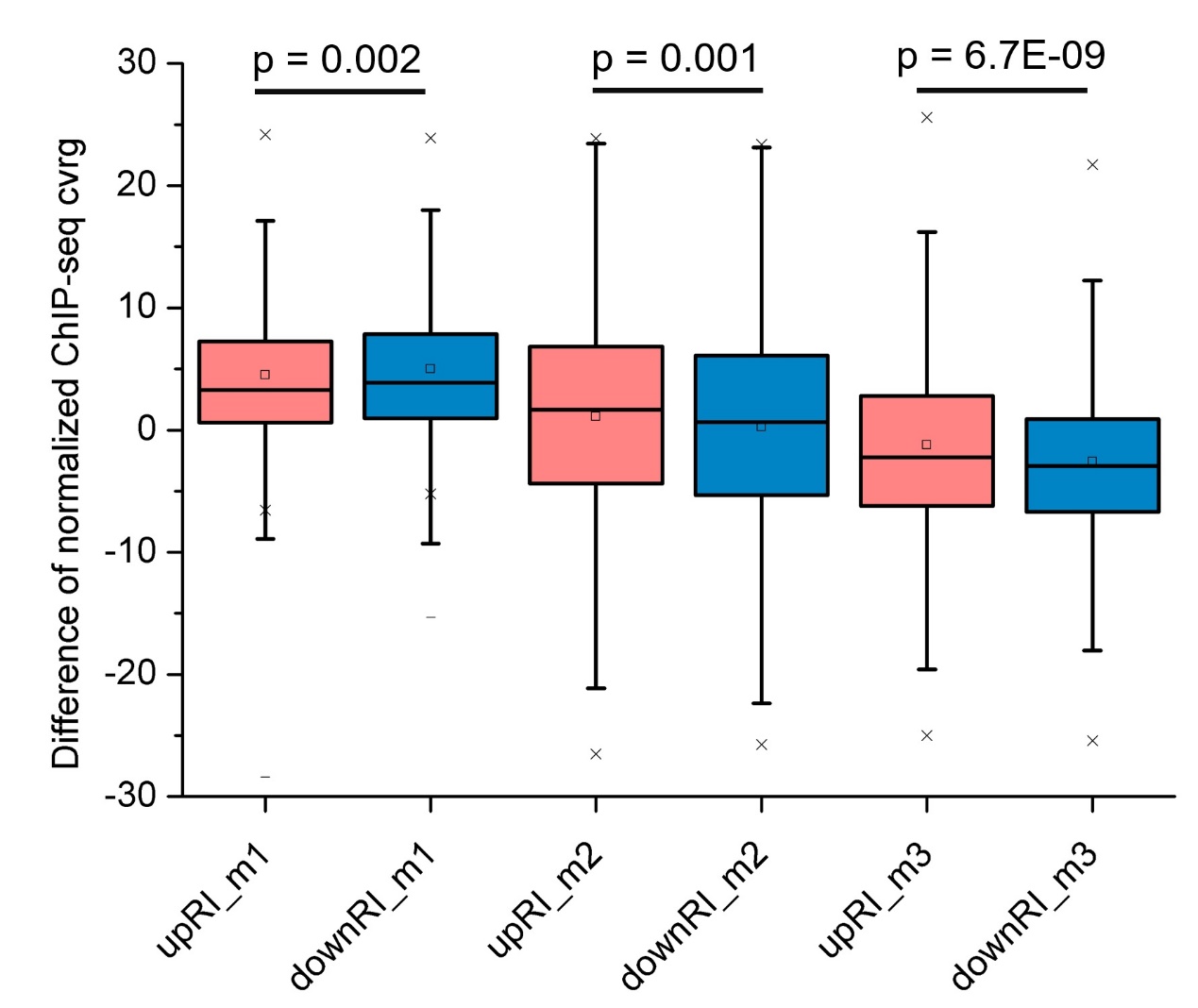


**Figure S6 - Box plots for levels of H3K36 methylations at IRI-up or IRI-down introns.**

upRI and downRI represent introns with increased or decreased intron retention index (IRI) to more than 2-fold in *725Ri-1* compared to WT rice, respectively. m1, m2 and m3 denote H3K36me1, H3K36me2 and H3K36me3, respectively. p value of t-test was shown.


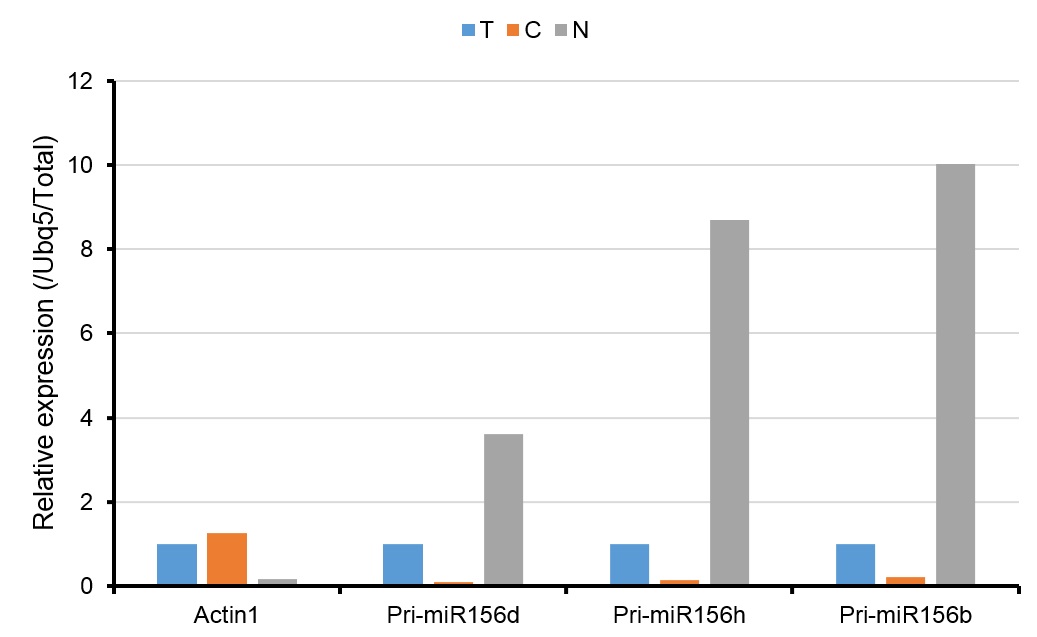


**Figure S7 - qPCR validation of quality of cellular fractionation in wild type rice.**

Total (T), cytoplasmic (C) and nuclear (N) RNAs were quantified using cytoplasmic enriched gene *Actin1* and nuclear enriched primary microRNAs (Pri-miR156d, Pri-miR156h and Pri-miR156b). Cellular fractionation method and enriched genes were based on previous publications [33, 34].


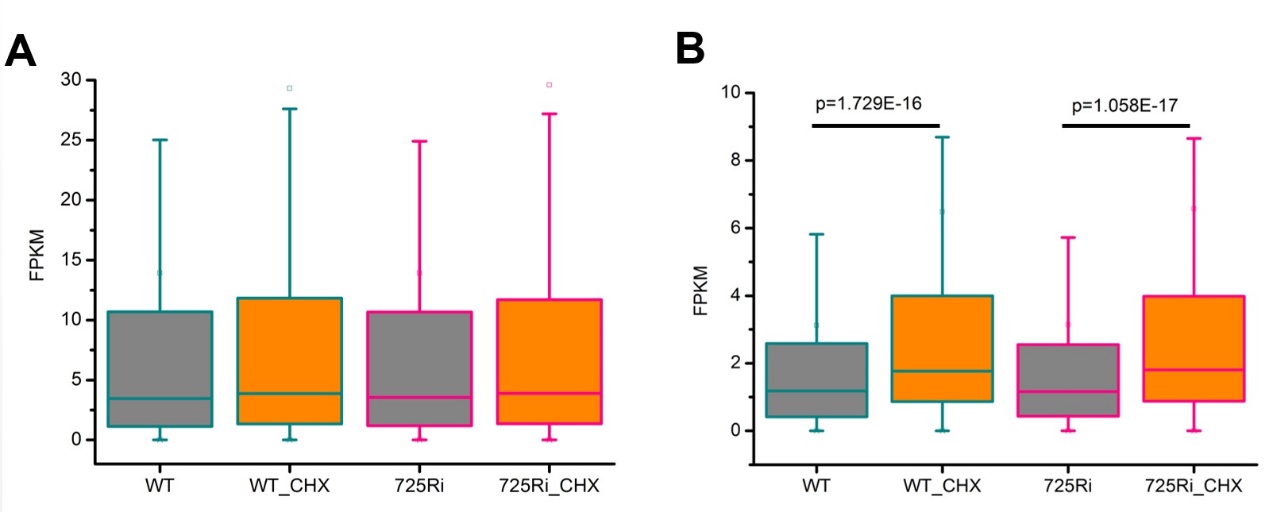


**Figure S8 - Box plot for expression level of transcripts with premature termination codon.**

Both wild-type (WT) and mutant (725Ri) rice samples were treated with CHX were analyzed based on newly generated RNA-seq data. P values were based on paired *t*-test.


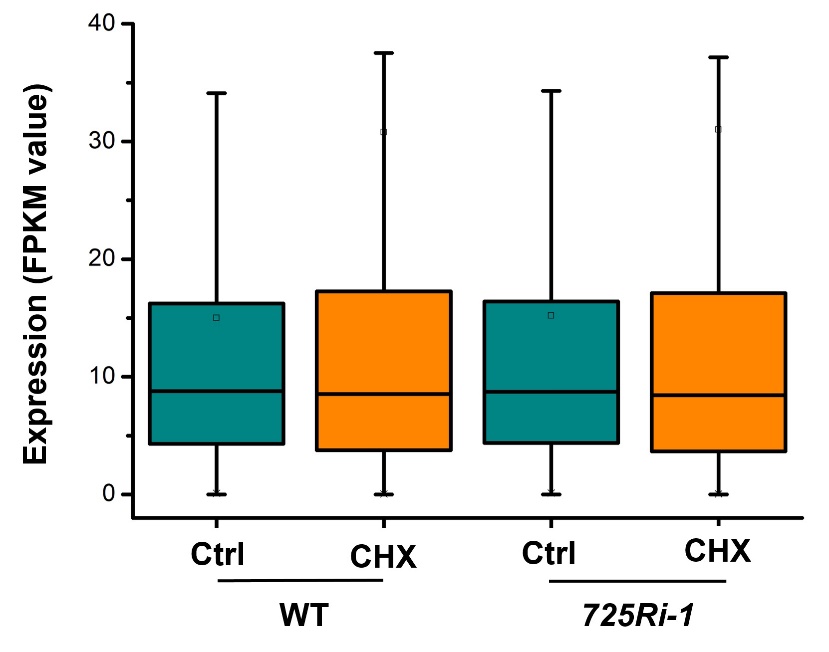


**Figure S9 - Box plot of gene expression before and after cycloheximide (CHX) treatment in either wild-type or 725Ri-1 rice.**

Only genes with 2-fold changes of IRI were used for analysis.


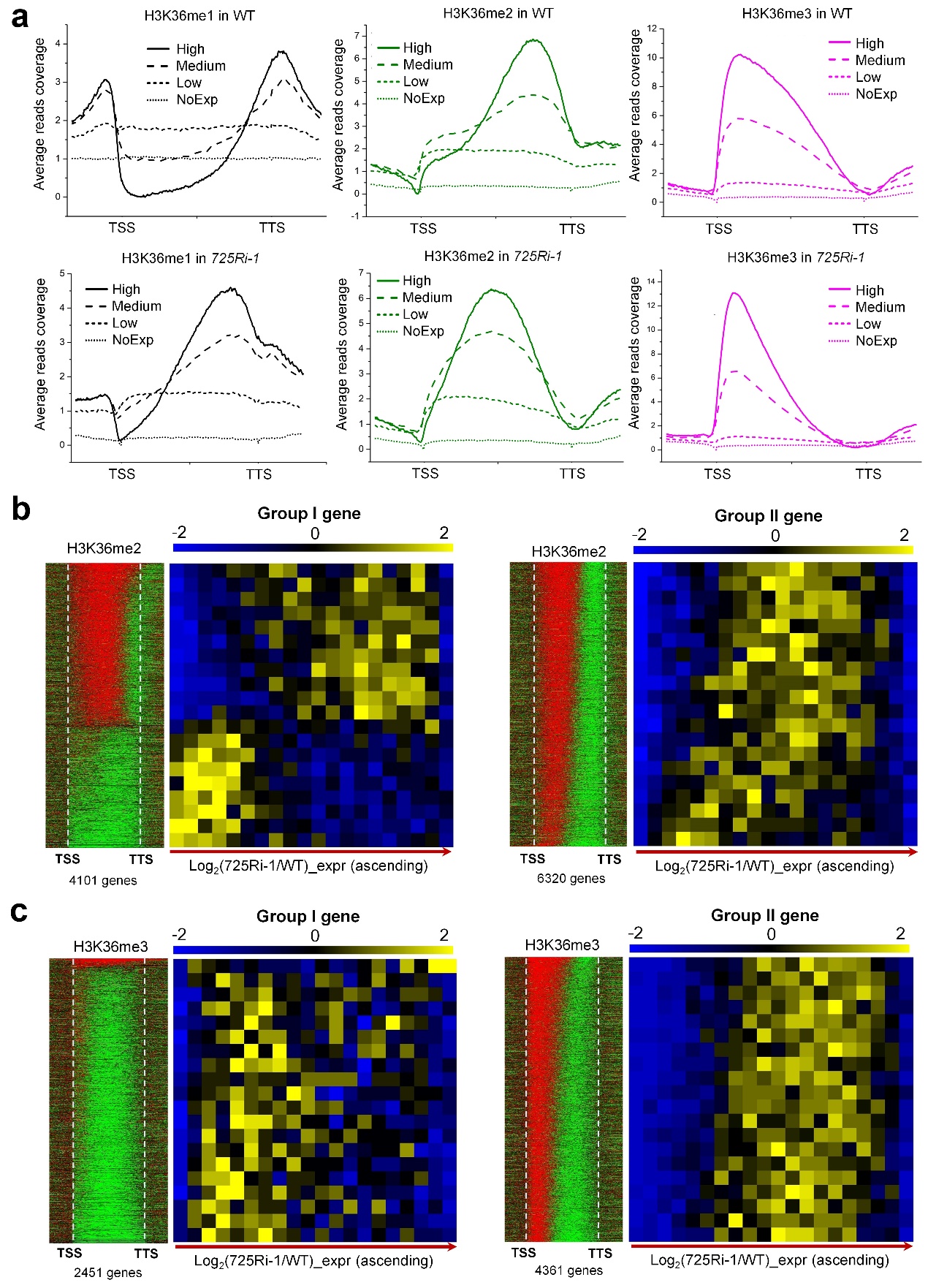


**Figure S10 - H3K36 methylations associate with gene expression levels.**

**(a)** Levels of H3K36 methylations positively correlate with gene expression levels in both WT (upper panel) and *725Ri-1* mutant rice (bottom panel). High, Medium, Low, NoExp represent expression levels of FPKM ≥ 10, FPKM between 1 and 10, FPKM between 0 and 1, FPKM = 0, respectively. Each plot was drawn in a similar way as described in Figure 3b. **(b-c)** Ranking the levels of H3K36 methylations (y axis) with the fold changes of gene expression (x axis) between *725Ri-1* and WT. The group I genes display more than 2-fold changes (increase or decrease) in the overall levels of H3K36me2 (b) or H3K36me3 (c) across gene body without obvious H3K36me2/me3 shifts in *725Ri-1* compared to the wild type. Group II genes show apparent H3K36me2/me3 shifts without obvious changes (≤ 2-fold) in total levels of H3K36me2 (b) or H3K36me3 (c).


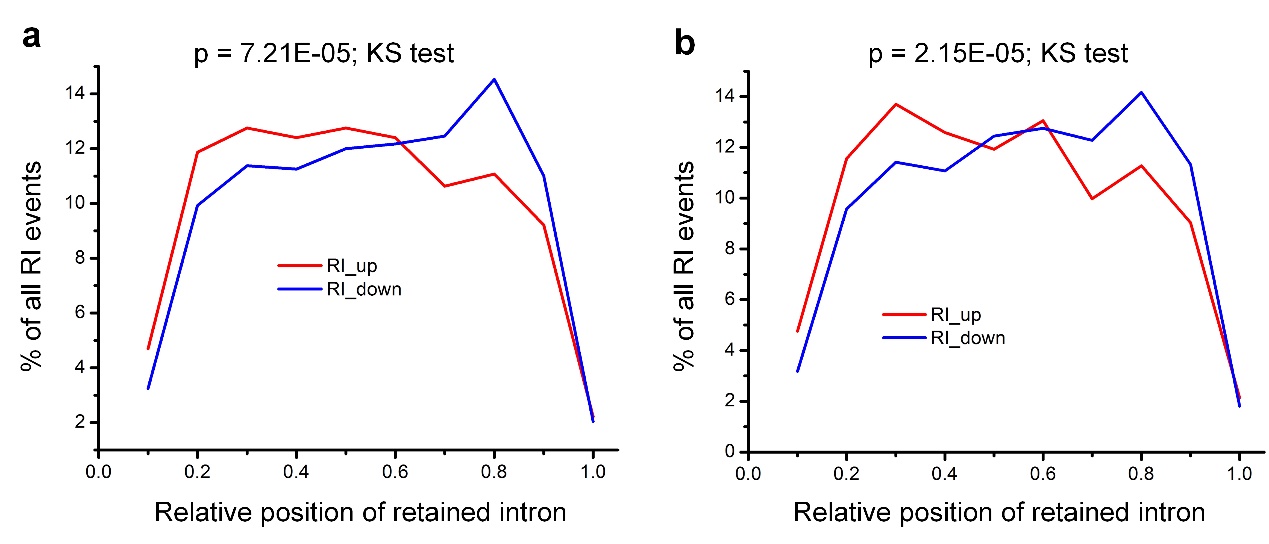


**Figure S11 - Distribution of up-regulated (red) and down-regulated (blue) intron retention (IR) events in genes with no obvious changes (≤ 2-fold) in total levels of H3K36me2 (a) or H3K36me3 (b).**


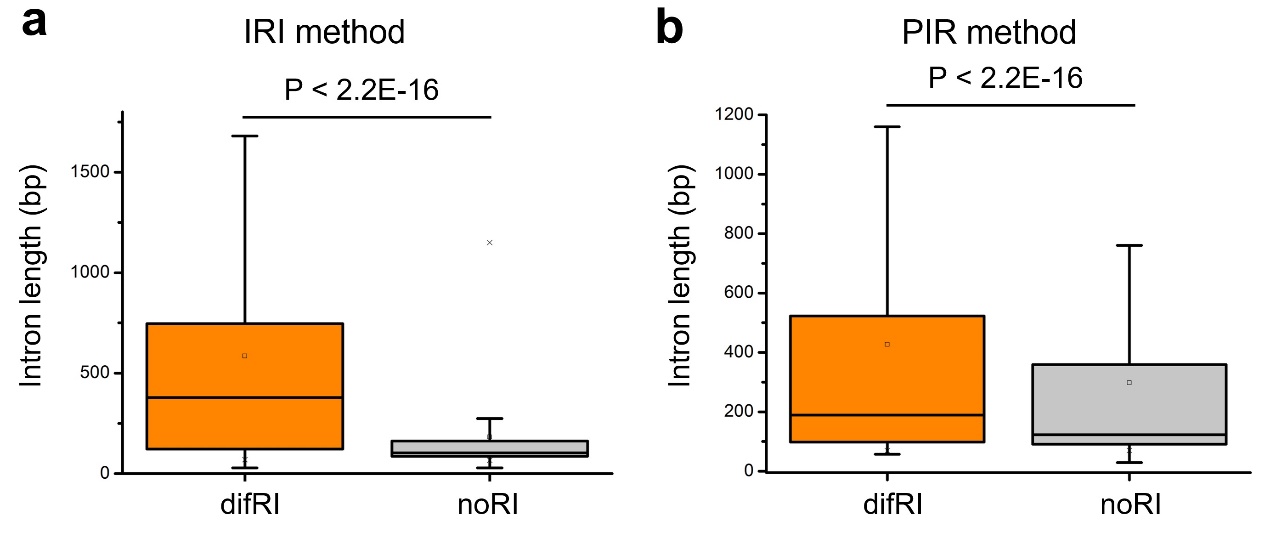


**Figure S12 - Box plot for intron length in two groups of introns by distinct methods in calculating the degree of intron retention.**

**(a)** Retained introns obtained by the IRI method [3]; **(b)** Retained introns obtained by the PIR method [2]. difRI represents introns that show 2-fold changes between *725Ri-1* and WT while noRI denotes introns that always have been spliced in both *725Ri-1* and WT. P value of KS-test between two groups was shown.


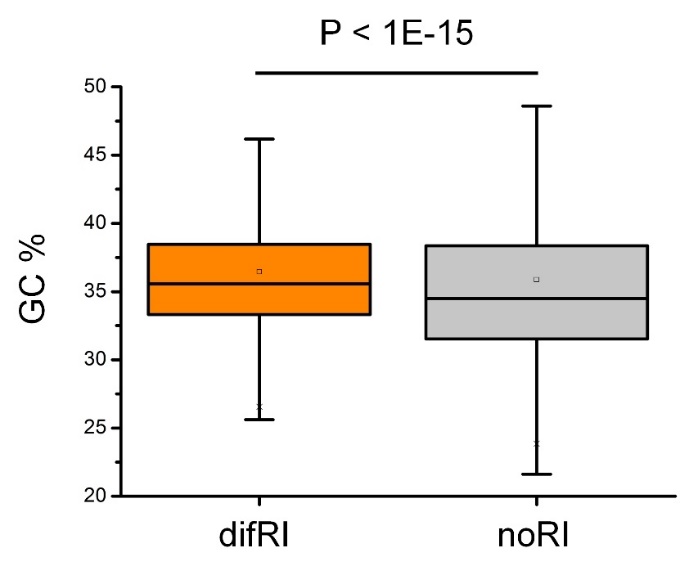


**Figure S13 - Box plot for GC percentage in two groups of introns.** difRI presents introns with at least 2-fold change of IRI between *725Ri-1* and WT while noRI denotes introns that always have been spliced in both *725Ri-1* and WT. P value of KS-test between two groups was shown.


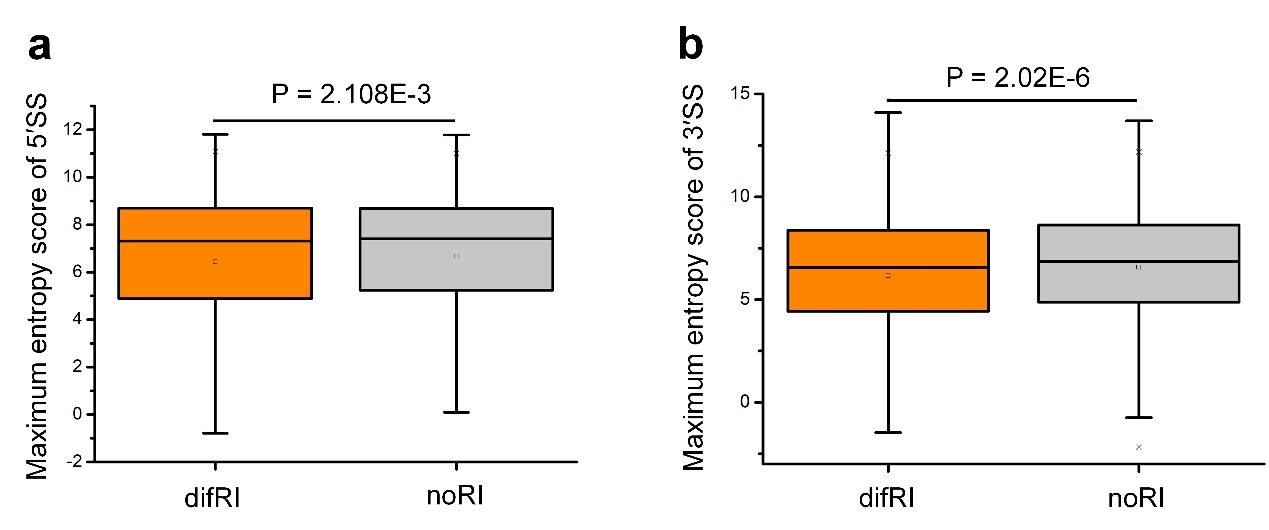


**Figure S14 - Box plot for maximum entropy score of 5′ splice site (a) and 3′ splice site (b) in two groups of introns.**

The maximum entropy score is calculated by MaxEntScan [37]. difRI presents introns with at least 2-fold change of IRI between *725Ri-1* and WT while noRI denotes introns that always have been spliced in both *725Ri-1* and WT. P value of KS-test between two groups was shown.


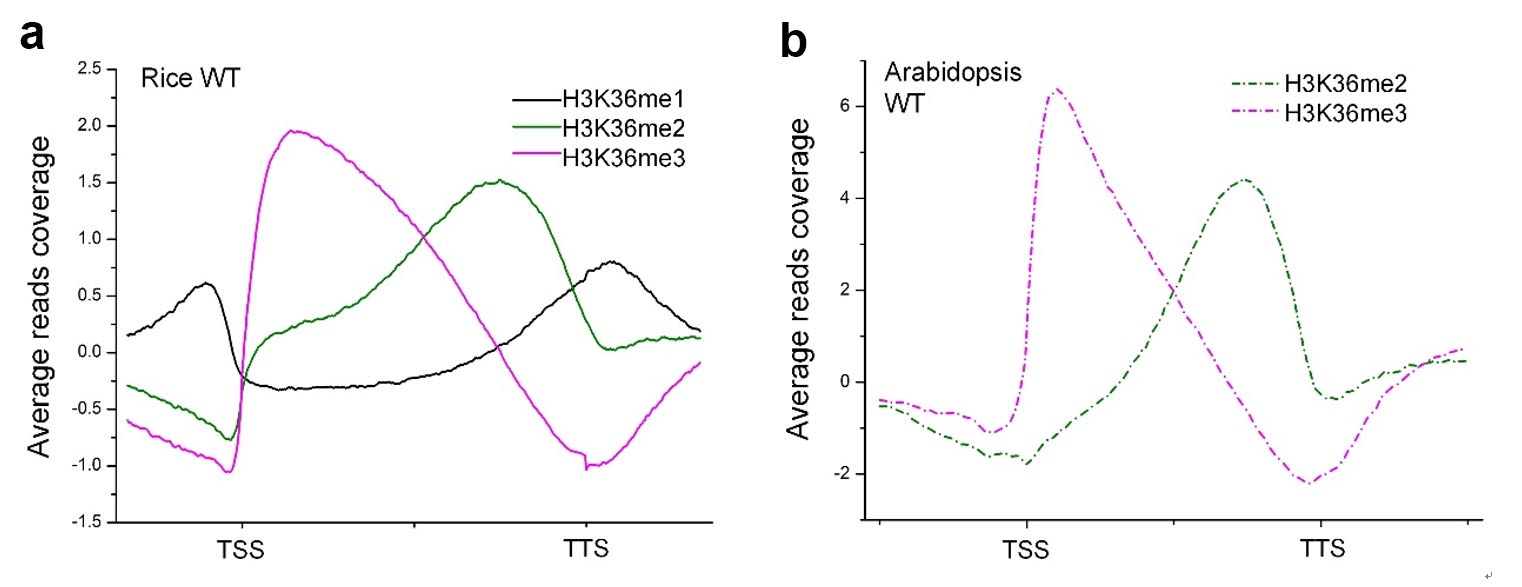


**Figure S15 - Comparison of H3K36me2/3 distribution across gene body between rice (a) and Arabidopsis (b).**

The x axis denotes the relative position of a gene, which include the gene body, the upstream and downstream 2 kb of a gene. TSS stands for transcription start site and TTS denotes transcription termination site. The y axis denotes the normalized coverage of ChIP-seq reads calibrated by the input sample. Arabidopsis ChIP-seq data sets were downloaded from published paper [37] and plotted in the same way.
