# Supplementary material for: Position-specific intron retention is mediated by the histone methyltransferase SDG725
Source: BMC Biol. 2018 Apr 30;16:44. doi: 10.1186/s12915-018-0513-8 (PMC5925840; doi:10.1186/s12915-018-0513-8)
Supplement: Supplementary file 2 — Table S1. Summary of the RNA-seq libraries. Table S2. Alternative splicing events discovered between rice RNA-seq data sets in this study compared with annotation by using SplAdder. Table S3. Primers used for validating intron-retention events. Table S4. The numbers of IRI changed introns and genes. Table S5. Summary of the ChIP-seq libraries. Table S6. Primers used to validate intron retention in the same gene by qRT-PCR and those used to validate H3K36me2 by ChIP-PCR. Table S7. Number of retained introns for previously identified IRI changed introns in nucleus or cytoplasm. Table S8. Expression changes of annotated rice splicing factors in our data. Table S9. Detailed information for ChIP-seq and RNA-seq data sets. (DOCX 43 kb) [file 12915_2018_513_MOESM2_ESM.docx]

**Position-specific intron retention is mediated by the histone methyltransferase SDG725**

Gang Wei, Kunpeng Liu, Ting Shen, Jinlei Shi, Bing Liu, Wenjing Yang, Miao Han, Maolin Peng, Haihui Fu, Yifan Song, Jun Zhu, Aiwu Dong, Ting Ni

**Supporting Tables**

**Table S1** - Summary of the RNA-seq libraries.

**Table S2** - Alternative splicing events discovered between rice RNA-seq datasets in this study compared with annotation by using SplAdder.

**Table S3** - Primers used for validating intron-retention events.

**Table S4** - The numbers of IRI changed introns and genes.

**Table S5** - Summary of the ChIP-seq libraries.

**Table S6** - Primers used to validate intron retention in the same gene by qRT-PCR and those used to validate the H3K36me2 by ChIP-PCR.

**Table S7.** Number of retained introns for previously identified IRI changed introns in nucleus or cytoplasm.

**Table S8** - Expression changes of annotated rice splicing factors in our data.

**Table S9** - Detailed information for ChIP-seq and RNA-seq data sets.

**Table S1 - Summary of the RNA-seq libraries.**

| **Sample** |  | **Mapped reads** | **Uniquely mapped reads** | **% of uniquely mapped** |
| --- | --- | --- | --- | --- |
| WT_rep1 | Reads_1 | 26,981,435 | 25,526,095 | 82.5 |
|  | Reads_2 | 26,984,354 | 25,432,448 | 82.2 |
| WT_rep2 | Reads_1 | 42,816,759 | 40,550,052 | 82.2 |
|  | Reads_2 | 43,344,874 | 40,787,553 | 82.7 |
| 725Ri_1_rep1 | Reads_1 | 58,648,715 | 55,476,580 | 84.1 |
|  | Reads_2 | 58,637,721 | 55,347,053 | 83.9 |
| 725Ri-1_rep2 | Reads_1 | 49,070,994 | 46,542,673 | 83.5 |
|  | Reads_2 | 49,652,774 | 46,856,670 | 84.1 |

**Table S2 - Alternative splicing events discovered between rice RNA-seq datasets in this study compared with annotation by using SplAdder.**

| **Types of alternative splicing** | **# of events** | **% of events** | **# of genes** |
| --- | --- | --- | --- |
| Skipped exon (SE) | 3,392 | 11.5% | 1,429 |
| Alternative 5′ splice site (A5SS) | 4,216 | 14.3% | 2,123 |
| Alternative 3′ splice site (A3SS) | 8,313 | 28.2% | 3,201 |
| Mutually exclusive exons (MXE) | 133 | 0.45% | 63 |
| Retained intron (RI) | 13,385 | 45.5% | 4,065 |

**Table S3 - Primers used for validating intron-retention events.**

|  | **Gene symbol** | **Primer pair*** | **Forward sequence (5´ to 3´)** | **Reverse sequence (5´ to 3´)** |
| --- | --- | --- | --- | --- |
| IRI up in *725Ri-1* | LOC_Os07g04990 | FE+RE | AGTGGGACGACAGGAGACTG | agcctcctgcagtctcctgt |
|  |  | FI+RI | CTTAACATGTCCCTATCATTG | agcctcctgcagtctcctgt |
|  | LOC_Os08g29150 | FE+RE | GCAGACAGTGTTATTCTTGAG | cgctgtaccataaacttctg |
|  |  | FI+RI | GCGGCGGTTGGCGGCGGCGC | gaacaattcgatggattgat |
|  | LOC_Os08g10300 | FE+RE | CTAGCTAACTTAAACCTGATGC | tcgatattttctgatgcctt |
|  |  | FI+RI | TTCTGTAAAGGGGCAGACAG | attggctgacccagtttaca |
|  | LOC_Os05g48040 | FE+RE | CAGCCTCCTCGTTGGAATGG | ctaatccacttgccattccaac |
|  |  | FI+RI | GCACAGATGGTGAGCTAGTC | ctaatccacttgccattccaac |
|  | LOC_Os04g48770 | FE+RE | GCAACTGCTGAAGATGAAAATG | aaccgaaaacaagctttcct |
|  |  | FI+RI | AAGTGAGGCAACTGCTGAAG | ggagtagatgaaaatactag |
|  | LOC_Os03g02920 | FE+RE | GCTTCGTCAGGGGGTGCGAC | cagcgcgagggcgtcccggg |
|  |  | FI+RI | TCCACGACTGCTTCGTCAGG | gccgccgcatatatgcagtt |
|  | LOC_Os04g20270 | FE+RE | TGCCGCATCAGATCCGCCTG | aggagagtcgaagatctcag |
|  |  | FI+RI | TGTCGAATTTGCGTTAACCCT | aggagagtcgaagatctcag |
|  | LOC_Os08g36220 | FE+RE | TTCCGAGACCGGGCGTTCGG | ctcctcgtcgtcctccatc |
|  |  | FI+RI | GTTTCTTTGCTTGCGGTGTTG | ctcctcgtcgtcctccatc |
|  | LOC_Os02g02480 | FE+RE | CTGCGATGGAGGACTTGGGA | ctgacatcatcaatcccaag |
|  |  | FI+RI | GTTGCCAACTACTTGCCATAG | ctgacatcatcaatcccaag |
|  | LOC_Os11g13810 | FE+RE | TCAATTTTCTGCGTTCATTT | tttggcaaatctggtttccc |
|  |  | FI+RI | TTCAGGAAGTATTGGAAGGA | tttctcttcttcacttttgt |
| IRI down in *725Ri-1* | LOC_Os05g50360 | FE+RE | CCAAGACCGAATCATGTTCC | ggtgcacatcatgcgagactg |
|  |  | FI+RI | CAAGATATATGGGCCAAGACC | acagctatttaggggtggttg |
|  | OC_Os05g44350 | FE+RE | CGATTGAGGTAGATGACGAG | ctggctgcaagtaaacaggct |
|  |  | FI+RI | CTGCCTCTGAACCCGATTGAG | gattaccatgaatcagaaaac |
|  | LOC_Os10g42690 | FE+RE | CTGAAGCAATTGTCTGTGCT | cagctgaagcattacaagaacc |
|  |  | FI+RI | CAGGAGCAACTGCTGCTGATG | gaacattaacccaacagaaag |
|  | LOC_Os06g49080 | FE+RE | GGAGTTTCGAGCGTATTTGG | cagattcctcaaacatcacac |
|  |  | FI+RI | CTTTTGGTCATCCAAGCTCTG | catctttcttccatgtaacag |
|  | LOC_Os11g32810 | FE+RE | CTTATTGTGCATGTATGCAAC | cataaatatatttccactcgg |
|  |  | FI+RI | TTGGAATCACTTATTGTGCAT | ctaaaccaataaaagcacagg |
|  | LOC_Os04g45970 | FE+RE | GGTGGAGTGCTCAACAGGGA | ctcgtcagcttctggatcagt |
|  |  | FI+RI | GAAAGATTTTCATGATGCGGA | aggtataatgggtgaaaatgc |
|  | LOC_Os03g55490 | FE+RE | TTACCGAGGTTCTTGGAACAG | cttccaacaagtctttcaagc |
|  |  | FI+RI | ATATTCCGAGTCGACCCTTTC | cagtaggtagctaaagcatttc |
|  | LOC_Os09g31130 | FE+RE | GTTTTTGGGGGTTTGATTGTC | ccccaacttcgccgtggaag |
|  |  | FI+RI | GTCCTATGGCTTTTGCAGAGA | tcaggcatctcttggagaag |
|  | LOC_Os02g46990 | FE+RE | CTTGTGCGAACGGAACAAGAC | tatgtacttgtccagatatag |
|  |  | FI+RI | AGCTGATGCTTTCTTGTGCGA | gtgattgattgattgcaaatc |
|  | LOC_Os02g36340 | FE+RE | CATTGGTGCACAGATATTAC | accattgctgcccgtctgatg |
|  |  | FI+RI | GAATATGGCATTGGTGCACAG | actgatggattctgtaattag |

* FE: forward exon-spanning primer; RE: reverse downstream exon primer paired with FE to detect spliced signal. FI: forward intronic primer; RI: reverse downstream primer paired with FI to detect unspliced signal.

**Table S4 - The numbers of IRI changed introns and genes.**

| *725Ri-1* vs. WT | | *708Ri-1* vs. WT | |
| --- | --- | --- | --- |
| IRI up 2-fold | 2089 introns | IRI up 2-fold | 3326 introns |
|  | 1806 genes |  | 2861 genes |
| IRI down 2-fold | 4214 introns | IRI down 2-fold | 2497 introns |
|  | 3315 genes |  | 2212 genes |

**Table S5 - Summary of the ChIP-seq libraries.**

| Sample | # of raw reads | # of mapped reads | # of uniquely mapped reads | # of uniquely mapped non-redundant reads |
| --- | --- | --- | --- | --- |
| 708Ri-1_input | 16,979,399 | 16,435,424 | 12,553,791 | 12,277,438 |
| 708Ri-1_me1 | 13,421,231 | 9,134,257 | 7,749,542 | 7,145,737 |
| 708Ri-1_me2 | 24,484,392 | 16,688,289 | 14,949,432 | 12,561,644 |
| 708Ri-1_me3 | 12,236,193 | 10,473,112 | 9,506,109 | 8,770,630 |
| 725Ri-1_input | 16,903,558 | 16,476,241 | 12,393,556 | 12,068,343 |
| 725Ri-1_me1 | 13,915,986 | 10,599,522 | 9,542,416 | 9,039,882 |
| 725Ri-1_me2 | 11,726,317 | 9,622,130 | 8,695,067 | 7,995,205 |
| 725Ri-1_me3 | 27,012,153 | 19,996,332 | 18,175,456 | 14,843,008 |
| WT_input | 18,399,971 | 17,483,048 | 13,390,056 | 12,894,748 |
| WT_me1 | 12,956,355 | 10,305,210 | 9,063,821 | 8,445,822 |
| WT_me2 | 14,479,523 | 9,797,437 | 8,983,436 | 8,301,191 |
| WT_me3 | 13,631,425 | 11,153,934 | 10,408,174 | 9,534,079 |

**Table S6 - Primers used to validate intron retention in the same gene by qRT-PCR and those used to validate the H3K36me2 by ChIP-PCR.**

| **Type** | **Gene symbol** | **Primer name*** | **Primer sequence (5´ to 3´)** |
| --- | --- | --- | --- |
| qRT-PCR | LOC_Os01g24680 | F-E-Up | TCTCCATCGATGTATTGCTA |
|  |  | F-I-Up | AGGAGTTAACAGTACACTTC |
|  |  | R-Up | ctgtaacaacaatagctttc |
|  |  | F-E-Down | GTTCCTCTGAGTGCACCAGC |
|  |  | F-I-Down | GCATCCAGATATACAGCATC |
|  |  | R-Down | catacagattacacataactc |
|  | LOC_Os08g01760 | F-E-Up | ACTGTGTTAAGGGCCAAGAA |
|  |  | F-I-Up | GAGAGGTTGCTTTGCTTTCT |
|  |  | R-Up | ttgctacgaaggaataatcg |
|  |  | F-E-Down | CCGACAGGTGAAGATCATTG |
|  |  | F-I-Down | GTTGTTCTGTTGATTCACCA |
|  |  | R-Down | acggtcaagatcctcgtagg |
| ChIP-PCR | LOC_Os01g24680 | F1 | TGACTGGAGAGTGGGGGAAT |
|  |  | R1 | AATTGAGCCCCAACCCACAA |
|  |  | F2 | ATGCTGAAGCTCTTCGGAGG |
|  |  | R2 | GCCACCCTGAACACTACCAA |
|  |  | F3 | CCGCGTCTTGTTGGATTGAC |
|  |  | R3 | GACCCAATTGGTGTGCCTCT |
|  |  | F4 | TGCAGAGTCGTGTGAGGAAA |
|  |  | R4 | AACAGCCTGCAACGAACAAT |
|  |  | F5 | GCCTCTCGGAAGGGCTTTTA |
|  |  | R5 | TACCAAGCACCACCCACAAA |
|  |  | F6 | CTAAGCAAGTCAAGGCCCGA |
|  |  | R6 | CCCCATTGGCGTGTGTTCTA |
|  | LOC_Os08g01760 | F1 | AGTTGAGTTGAGAGTTGCTGACA |
|  |  | R1 | TGCAATGATGCGACCTTTGG |
|  |  | F2 | GCATGAGATCACTGGGGAGG |
|  |  | R2 | AGGGCATTATGAAGGCACCC |
|  |  | F3 | ACCGTGCTAAAGGAACGCTA |
|  |  | R3 | ATCGCCGACTCTGTGTATGG |
|  |  | F4 | GAAATGCGTGCTGGGGATTC |
|  |  | R4 | TGGGTTGCTCCAAGTGTTCT |
|  |  | F5 | GTGCGCCAAAAGTGTACGAG |
|  |  | R5 | GTAGCTCAAATGGCTTTGCTGT |
|  |  | F6 | TCAAGCTTGCAGAGAGTGGG |
|  |  | R6 | CCCAGGCTATGAACCACAGA |

* F-E-Up (forward exon-spanning primer) paired with R-Up (reverse downstream exon primer) to detect spliced signal in *725Ri-1* IRI-up event.

F-I-Up (forward intronic primer) paired with R-Up (reverse downstream exon primer) to detect unspliced signal in *725Ri-1* IRI-up event.

F-E-Down (forward exon-spanning primer) paired with R-Down (reverse downstream exon primer) to detect spliced signal in *725Ri-1* IRI-down event.

F-I-Down (forward intronic primer) paired with R-Down (reverse downstream exon primer) to detect unspliced signal in *725Ri-1* IRI-down event.

F1 paired with R1 to detect the ChIP signal that indicated in Figure 5. Number indicated the location of genes in Figure 5.

**Table S7. Number of retained introns for previously identified IRI changed introns in nucleus or cytoplasm.**

|  | **Sample** | **Nucleus** | **Cytoplasm** | **Shared** |
| --- | --- | --- | --- | --- |
| **upRIs** | WT | 1,515 | 273 | 254 |
|  | *725Ri-1* | 1,617 | 362 | 351 |
| **downRIs** | WT | 3,248 | 629 | 612 |
|  | *725Ri-1* | 3,408 | 750 | 731 |

**Table S8. Expression changes of annotated rice splicing factors in our data.**

| **Gene_ID** | **WT**  **(FPKM value)** | ***725Ri-1***  **(FPKM value)** | **Log_2_**  **(Fold-Change)** | | ***P* value** |
| --- | --- | --- | --- | --- | --- |
| LOC_Os01g15860 | 1.608 | 2.812 | 0.806 | 0.002 | |
| LOC_Os02g01250 | 23.835 | 26.159 | 0.134 | 0.572 | |
| LOC_Os02g03440 | 2.329 | 2.545 | 0.128 | 0.828 | |
| LOC_Os02g30624 | 40.974 | 42.879 | 0.066 | 0.889 | |
| LOC_Os02g37430 | 112.851 | 108.630 | -0.055 | 0.796 | |
| LOC_Os03g29740 | 5.617 | 7.786 | 0.471 | 0.195 | |
| LOC_Os04g39444 | 9.012 | 9.275 | 0.042 | 0.881 | |
| LOC_Os05g24970 | 38.360 | 43.674 | 0.187 | 0.389 | |
| LOC_Os05g32310 | 24.944 | 24.549 | -0.023 | 0.922 | |
| LOC_Os05g51650 | 14.000 | 10.579 | -0.404 | 0.523 | |
| LOC_Os06g04300 | 16.895 | 19.155 | 0.181 | 0.429 | |
| LOC_Os06g42770 | 1.290 | 1.871 | 0.537 | 0.076 | |
| LOC_Os07g10256 | 23.954 | 22.385 | -0.098 | 0.667 | |
| LOC_Os07g38420 | 21.250 | 23.492 | 0.145 | 0.498 | |
| LOC_Os07g41790 | 37.815 | 48.201 | 0.350 | 0.121 | |
| LOC_Os08g08040 | 42.232 | 46.100 | 0.126 | 0.566 | |
| LOC_Os10g34520 | 113.345 | 92.218 | -0.298 | 0.163 | |
| LOC_Os10g35550 | 14.695 | 15.405 | 0.068 | 0.744 | |
| LOC_Os10g41790 | 3.117 | 3.501 | 0.168 | 0.514 | |
| LOC_Os11g43620 | 7.248 | 8.243 | 0.185 | 0.556 | |

We utilized the rice gene annotation database in gramene (http://www.gramene.org/), where gene ontology (GO) term was used to annotated rice genes. We extracted 20 genes with GO terms containing “splicing” or “splicesome”, and examined their expression changes in our RNA-seq data. According to the criteria used to define differential expressed genes (FPKM > 1, 2-fold change and P value < 0.01), none annotated splicing factors was differentially expressed (see Table S8 above).

**Table S9 - Detailed information for ChIP-seq and RNA-seq data sets.**

| Lib name | Source name | Organism | Library type | SRA number |
| --- | --- | --- | --- | --- |
| Lib_1 | SDG725-Ri-1_input | rice (Nipponbare) | ChIP-seq | Data generated by this study |
| Lib_2 | SDG725-Ri-1_H3K36me1 | rice (Nipponbare) | ChIP-seq | Data generated by this study |
| Lib_3 | SDG725-Ri-1_H3K36me2 | rice (Nipponbare) | ChIP-seq | Data generated by this study |
| Lib_4 | SDG725-Ri-1_H3K36me3 | rice (Nipponbare) | ChIP-seq | Data generated by this study |
| Lib_5 | WT_input | rice (Nipponbare) | ChIP-seq | SRP063912 |
| Lib_6 | WT_H3K36me1 | rice (Nipponbare) | ChIP-seq | SRP063912 |
| Lib_7 | WT_H3K36me1 | rice (Nipponbare) | ChIP-seq | SRP063912 |
| Lib_8 | WT_H3K36me1 | rice (Nipponbare) | ChIP-seq | SRP063912 |
| Lib_9 | SDG708-Ri-1_input | rice (Nipponbare) | ChIP-seq | SRP063912 |
| Lib_10 | SDG708-Ri-1_H3K36me1 | rice (Nipponbare) | ChIP-seq | SRP063912 |
| Lib_11 | SDG708-Ri-1_H3K36me2 | rice (Nipponbare) | ChIP-seq | SRP063912 |
| Lib_12 | SDG708-Ri-1_H3K36me3 | rice (Nipponbare) | ChIP-seq | [SRP063912](http://www.ncbi.nlm.nih.gov/Traces/sra_sub/?acc=SRP063912&focus=SRP063912&from=list&action=show:STUDY) |
| Lib_13 | SDG725-Ri-1_rep1 | rice (Nipponbare) | RNA-seq | Data generated by this study |
| Lib_14 | SDG725-Ri-1_rep2 | rice (Nipponbare) | RNA-seq | Data generated by this study |
| Lib_15 | WT_rep1_pairing_SDG725 | rice (Nipponbare) | RNA-seq | Data generated by this study |
| Lib_16 | WT_rep2_pairing_SDG725 | rice (Nipponbare) | RNA-seq | Data generated by this study |
| Lib_17 | SDG708-Ri-1_rep1 | rice (Nipponbare) | RNA-seq | [SRP063912](http://www.ncbi.nlm.nih.gov/Traces/sra_sub/?acc=SRP063912&focus=SRP063912&from=list&action=show:STUDY) |
| Lib_18 | SDG708-Ri-1_rep2 | rice (Nipponbare) | RNA-seq | [SRP063912](http://www.ncbi.nlm.nih.gov/Traces/sra_sub/?acc=SRP063912&focus=SRP063912&from=list&action=show:STUDY) |
| Lib_19 | WT_rep1_pairing_SDG708 | rice (Nipponbare) | RNA-seq | [SRP063912](http://www.ncbi.nlm.nih.gov/Traces/sra_sub/?acc=SRP063912&focus=SRP063912&from=list&action=show:STUDY) |
| Lib_20 | WT_rep2_pairing_SDG708 | rice (Nipponbare) | RNA-seq | [SRP063912](http://www.ncbi.nlm.nih.gov/Traces/sra_sub/?acc=SRP063912&focus=SRP063912&from=list&action=show:STUDY) |
